# Recessive genetic contribution to congenital heart disease in 5,424 probands

**DOI:** 10.1073/pnas.2419992122

**Published:** 2025-03-03

**Authors:** Weilai Dong, Sheng Chih Jin, Michael C. Sierant, Ziyu Lu, Boyang Li, Qiongshi Lu, Sarah U. Morton, Junhui Zhang, Francesc López-Giráldez, Carol Nelson-Williams, James R. Knight, Hongyu Zhao, Junyue Cao, Shrikant Mane, Peter J. Gruber, Monkol Lek, Elizabeth Goldmuntz, John Deanfield, Alessandro Giardini, Seema Mital, Mark Russell, J. William Gaynor, James F. Cnota, Michael Wagner, Deepak Srivastava, Daniel Bernstein, George A. Porter, Jane Newburger, Amy E. Roberts, Mark Yandell, H. Joseph Yost, Martin Tristani-Firouzi, Richard Kim, Jonathan Seidman, Wendy K. Chung, Bruce D. Gelb, Christine E. Seidman, Richard P. Lifton, Martina Brueckner

**Affiliations:** ^a^Department of Genetics, Yale School of Medicine, New Haven, CT 06510; ^b^Laboratory of Human Genetics and Genomics, The Rockefeller University, New York, NY 10065; ^c^Department of Genetics, Washington University School of Medicine, St. Louis, MO 63110; ^d^Department of Pediatrics, Washington University School of Medicine, St. Louis, MO 63110; ^e^Laboratory of Single-cell Genomics and Population Dynamics, The Rockefeller University, New York, NY 10065; ^f^Department of Biostatistics, Yale School of Public Health, New Haven, CT 06510; ^g^Department of Biostatistics & Medical Informatics, University of Wisconsin, Madison, WI 53706; ^h^Division of Newborn Medicine, Department of Pediatrics, Boston Children’s Hospital, Boston, MA 02115; ^i^Manton Center for Orphan Disease Research, Boston Children’s Hospital, Boston, MA 02115; ^j^Broad Institute of Massachusetts Institute of Technology and Harvard, Boston, MA 02142; ^k^Yale Center for Genome Analysis, Yale University, New Haven, CT 06516; ^l^Department of Surgery, Yale University School of Medicine, New Haven, CT 06510; ^m^Division of Cardiology, Children’s Hospital of Philadelphia, Department of Pediatrics, Perelman School of Medicine, University of Pennsylvania, Pennsylvania, PA 19104; ^n^Institute of Cardiovascular Science, University College London, London WC1E 6BT, United Kingdom; ^o^Pediatric Cardiology, Great Ormond Street Hospital, London WC1N 3JH, United Kingdom; ^p^Division of Cardiology, Department of Pediatrics, The Hospital for Sick Children, University of Toronto, Toronto, ON M5G1X8, Canada; ^q^Department of Pediatrics and Communicable Diseases, University of Michigan, Ann Arbor, MI 48109; ^r^Division of Cardiothoracic Surgery, Children’s Hospital of Philadelphia, Philadelphia, PA 19104; ^s^Division of Cardiology, Cincinnati Children’s Hospital Medical Center, Cincinnati, OH 45229; ^t^Division of Biomedical Informatics, Cincinnati Children’s Hospital Medical Center, Cincinnati, OH 45229; ^u^Division of Biostatistics and Epidemiology, Cincinnati Children’s Hospital Medical Center, Cincinnati, OH 45229; ^v^Gladstone Institute of Cardiovascular Disease, San Francisco, CA 94158; ^w^Department of Pediatrics, Cardiology, Stanford University, Stanford, CA 94304; ^x^Department of Pediatrics, The School of Medicine and Dentistry, University of Rochester Medical Center, Rochester, NY 14642; ^y^Department of Cardiology, Boston Children’s Hospital, Boston, MA 02115; ^z^Department of Human Genetics, University of Utah and School of Medicine, Salt Lake City, UT 84112; ^aa^The Catholic University of America, Washington, DC 20064; ^bb^Division of Pediatric Cardiology, University of Utah, Salt Lake City, UT 84112; ^cc^Pediatric Cardiac Surgery, Smidt Heart Institute, Cedars-Sinai Medical Center, Los Angeles, CA 90048; ^dd^Department of Genetics, Harvard Medical School, Boston, MA 02115; ^ee^Department of Pediatrics, Columbia University Medical Center, New York, NY 10032; ^ff^Department of Medicine, Columbia University Medical Center, New York, NY 10032; ^gg^Department of Pediatrics, Boston Children’s Hospital, Harvard Medical School, Boston, MA 02115; ^hh^Mindich Child Health and Development Institute, Icahn School of Medicine at Mount Sinai, New York, NY 10029; ^ii^Department of Pediatrics, Icahn School of Medicine at Mount Sinai, New York, NY 10029; ^jj^Cardiovascular Division, Brigham and Women’s Hospital, Boston, MA 02115; ^kk^HHMI, Chevy Chase, MD 20815; ^ll^Department of Pediatrics, Section of Cardiology, Yale School of Medicine, New Haven, CT 06510

**Keywords:** genomics, congenital heart disease, exome-sequencing, human genetics

## Abstract

Variants with large effect contribute to congenital heart disease (CHD). To date, recessive genotypes (RGs) have commonly been implicated through anecdotal ascertainment of consanguineous families and candidate gene-based analysis; the recessive contribution to the broad range of CHD phenotypes has been limited. We analyzed whole exome sequences of 5,424 CHD probands. Rare damaging RGs were estimated to contribute to at least 2.2% of CHD, with greater enrichment among laterality phenotypes (5.4%) versus other subsets (1.4%). Among 108 curated human recessive CHD genes, there were 66 RGs, with 54 in 11 genes with >1 RG, 12 genes with 1 RG, and 85 genes with zero. RGs were more prevalent among offspring of consanguineous union (4.7%, 32/675) than among nonconsanguineous probands (0.7%, 34/4749). Founder variants in *GDF1* and *PLD1* accounted for 74% of the contribution of RGs among 410 Ashkenazi Jewish probands. We identified genome-wide significant enrichment of RGs in *C1orf127*, encoding a likely secreted protein expressed in embryonic mouse notochord and associated with laterality defects. Single-cell transcriptomes from gastrulation-stage mouse embryos revealed enrichment of RGs in genes highly expressed in the cardiomyocyte lineage, including contractility-related genes *MYH6, UNC45B*, *MYO18B*, and *MYBPC3* in probands with left-sided CHD, consistent with abnormal contractile function contributing to these malformations. Genes with significant RG burden account for 1.3% of probands, more than half the inferred total. These results reveal the recessive contribution to CHD, and indicate that many genes remain to be discovered, with each likely accounting for a very small fraction of the total.

## Significance

Understanding the genetic basis of congenital heart disease (CHD) provides insight into cardiac development, with implications for prevention, diagnosis, and treatment of the most common congenital disease. Exome sequencing of 5,424 CHD probands estimates the contribution of recessive genotypes (RGs) to be ~2.2%. *C1orf127* was identified as a recessive risk gene for laterality-associated CHD, and founder variants in *GDF1* and *PLD1* account for 74% of the estimated contribution from RGs in Ashkenazi Jews. Variants in known genes were increased among probands of consanguineous (4.7%), rather than nonconsanguineous union (0.7%) accounting for 62% of inferred total RG burden. Single-cell transcriptomes of mouse gastrulation implicated RGs affecting notochord- and cardiomyocyte lineage-expressed genes suggesting roles for cilia and cardiomyocyte contractility genes in CHD.

Congenital heart disease (CHD) comprises diverse structural malformations of the heart and great vessels (1). CHD accounts for about one-third of all birth defects with a global newborn incidence of 1 to 1.8% and prevalence of 0.16% across all ages (2–4). Surgical and catheter-based palliation and repair of structural cardiac malformations, along with improved medical management, have led to survival of almost 90% of CHD patients into adulthood, with 2.4 million people currently with CHD in the United States, including 1.4 million adults. Despite this significant progress, variable comorbidities including endocarditis, arrhythmia, reoperation, heart failure, pulmonary hypertension, and neurodevelopmental defects make CHD a frequently lifelong disease (5–9).

CHD has a strong genetic basis. About 25% of cases are associated with large chromosomal abnormalities or copy number variations (10). Environmental factors, such as teratogens, deficiency for essential nutrients, maternal diseases such as diabetes, and maternal intrapartum infections, are thought to contribute to another ~10% of cases (11). Families with rare variants segregating with diverse CHD phenotypes including atrial septal defect (ASD), aortic valve disease, and heterotaxy have been shown to account for a fraction of cases as well (12–15), and many well-characterized syndromes caused by single-gene variants are associated with CHD (10). Nonetheless, reasonable estimates of the genetic contribution to the most common forms of CHD, which most often occur sporadically, have only become possible over the last decade with the advent of next-generation DNA sequencing and the unbiased collection of CHD probands, along with parental samples.

Variants with large effect in coding regions and flanking splice sites have been implicated in ~9% of cases (16–19), and de novo mutations in noncoding sequences occur more frequently than expected in people with CHD in whom exome sequencing has not identified a likely cause (20).

Recessive contributions to CHD are frequently identified in offspring of consanguineous union (21–23), and in patients with cardiovascular laterality defects (24, 25). Our previous report of recessive contributions to CHD based on exome sequencing of 2,871 probands identified two genes with genome-wide significance (*GDF1* and *MYH6)* (19). While several genes have been individually associated with recessive cases of CHD (19, 24), the collective recessive contribution to the burden of CHD remains unknown. Genomic analyses of ever-larger cohorts are making it possible to define the genetic architecture and genotype–phenotype associations in diseases such as CHD and autism, which are characterized by large genetic contributions with high locus heterogeneity and variable phenotypic expression. For example, biallelic variants are estimated to contribute to 5% of autism, which shares genes and genetic architecture with CHD (26). Herein, we report the use of WES to systematically analyze the spectrum and global contribution of recessive genotypes (RGs) in 5,424 unrelated CHD individuals from the NHLBI Bench-to-Bassinet Program. The results advance the understanding of the genetic architecture underlying human CHD and its phenotypic subsets.

## Results

### Cohort Description and Exome Sequencing.

We studied 5,424 CHD probands, including 3,716 parent–offspring trios and 1,708 singleton probands from the NHLBI Bench-to-Bassinet Program [Pediatric Cardiac Genomics Consortium (PCGC) and Pediatric Heart Network] (*SI Appendix*, Table S1 and Dataset S1). Probands from the United States and the United Kingdom were recruited for the study, with the sole inclusion criterion of structural CHD (27). Controls were siblings of probands with autism in 1,798 trios from the Simons Simplex autism cohort as previously reported (19). Cardiac phenotypes in CHD probands were broadly classified into five subgroups: Heterotaxy (HTX, 9.9%), conotruncal defect (CTD, including tetralogy of Fallot, 33.9%), left ventricular obstructive lesion [LVO, including hypoplastic left heart syndrome (HLHS), 25.5%], D-transposition of the great arteries (D-TGA, 7.1%) and Other (including ASD, atrioventricular canal and other forms of CHD not part of the HTX, CTD, or LVO subgroups) (*SI Appendix*, Table S1). Laterality-associated defects included HTX and others with cardiac abnormalities associated with left–right abnormalities including D-TGA and double-outlet right ventricle (DORV).

WES of all probands and available parents of the CHD cohort and controls were performed (*SI Appendix*, Table S2), and variants were called as described previously (28). Principal component analysis (PCA) from WES showed that 71.4% of CHD probands were of European ancestry, 6.3% African American, 9.6% Mexican, 2.1% East Asian, and 3.9% South Asian (*SI Appendix*, Table S1). The control cohort was broadly similar in ancestry distribution (*SI Appendix*, Table S1). Homozygous and compound heterozygous variant genotypes were called and annotated as rare damaging RGs if both alleles had frequency < 10^−3^ in gnomAD and Bravo databases and were likely loss of function (LoF) or damaging missense (D-mis) as called by MetaSVM (*Materials and Methods*). For compound heterozygous variants in singletons, co-occurrent damaging variant pairs were called and then trans variants were determined by using the Variant co-occurrence tool in gnomAD (https://gnomad.broadinstitute.org/variant-cooccurrence). This yielded 180 inferred compound heterozygous (ICH) RGs in 1,708 singleton probands, compared to 425 compound heterozygous RGs in 3,716 proband-parent trios (Dataset S2).

### RGs in Known CHD Genes Are Highly Enriched in Probands from Consanguineous Union.

Among the 5,424 CHD probands we identified a total of 1,253 rare damaging RGs (biallelic homozygous or compound heterozygous genotypes, RGs) in CHD probands (0.2 RGs per proband); among 1,798 controls there were 229 RGs (0.1 RGs per proband) (Table 1 and Dataset S2). Homozygous damaging RGs comprised 52% of all RGs in CHD probands but 24% of controls, implying greater consanguinity in the CHD cohort. Indeed, the mean inbreeding coefficient (*F)* in CHD probands was 1.8 × 10^−3^, significantly higher than found in controls (2.5 × 10^−4^, *P* = 4.1 × 10^−14^ by the Kolmogorov–Smirnov test). From the distribution of *F* (*SI Appendix*, Fig. S1 and Table S3), 675 probands (12.4% if the total cohort) had inbreeding coefficients of the expected value from the union of 4th cousins (9.0 × 10^−4^) or closer. In contrast, only 80 controls (4.4%) had this level of inbreeding coefficients.

**Table 1.** Enrichment of damaging RGs in known human recessive CHD genes

	All 19,347 genes						108 known human recessive CHD genes							
	Hom		CompHet		RGs		Hom		CompHet		RGs			
Sample set	#	Rate	#	Rate	#	Rate	#	Rate	#	Rate	#	Rate	Enrichment	*P*-Value
5,424 Cases	648	1.2 × 10^−1^	605	1.1 × 10^−1^	1253	2.3 × 10^−1^	32	5.9 × 10^−3^	34	6.3 × 10^−3^	66	1.2 × 10^−2^	3.9	**3.5 × 10** ^**−20**^
675 Consanguineous Cases	537	8.0 × 10^−1^	91	1.3 × 10^−1^	628	9.3 × 10^−1^	28	4.1 × 10^−2^	4	5.9 × 10^−3^	32	4.7 × 10^−2^	4.7	**1.8 × 10** ^**−12**^
4,749 Nonconsanguineous Cases	111	2.3 × 10^−2^	514	1.1 × 10^−1^	625	1.3 × 10^−1^	4	8.4 × 10^−4^	30	6.3 × 10^−3^	34	7.2 × 10^−^^3^	3.5	**8.7 × 10** ^**−10**^
1,798 Controls	55	3.0 × 10^−2^	174	1.0 × 10^−1^	229	1.3 × 10^−1^	1	5.6 × 10^−4^	1	5.6 × 10^−4^	2	1.1 × 10^−3^	0.6	8.3 × 10^−1^

Distribution of different types of damaging RGs in cases and controls and the enrichment of RGs in known human recessive CHD genes. Rate indicates the number of genotypes per individual. The expected number of RGs was determined based on the fitted values from the polynomial regression model using the de novo mutability. *P* values were calculated using the one-tailed binomial test. *P* values exceeding the Bonferroni multiple-testing cutoff (0.05/[2 × 2] = 0.0125) were bolded. # Hom: number of damaging homozygotes; # CompHet: number of damaging compound-heterozygotes; # RGs: number of damaging RGs.

To determine whether RGs occurred in known recessive CHD genes more often than expected by chance, we compiled a list of 108 known human recessive CHD genes based on the OMIM database and literature search (*SI Appendix*, *Materials and Methods* for details and Dataset S3). The enrichment of RGs in known human CHD genes among all CHD cases was evaluated using a binomial test, in which the expected number of RGs was estimated from each gene’s de novo mutability (29) adjusted by a polynomial model that adjusts for variable levels of consanguinity (19). 66 damaging RGs were found in these known human recessive CHD genes and were 3.9-fold more frequent than expected (*P* < 10^−19^; Table 1 and Dataset S2). These RGs can account for 1.2% of all cases. In contrast, there were only 2 RGs in these genes in controls (0.1% of probands), with no enrichment compared to the expected value (enrichment = 0.6; Table 1).

While offspring of consanguineous union (4th cousin or closer) comprise only 12.4% of all CHD probands, these probands had 48.5% (32/66) of the damaging RGs in the set of known human recessive CHD genes, including 87.5% (28/32) of the homozygous RGs (Table 1 and *SI Appendix*, Table S3). 4.7% of probands (32/675) from consanguineous union had RGs in known CHD genes, versus 0.7% from nonconsanguineous union (34/4749) (6.5 – fold enrichment, Fisher’s exact test *P* = 6.1 × 10^−13^). As expected, the percentage of probands with RGs in known CHD genes declined with diminishing inbreeding coefficient, from 9.5% among probands with *F* ≥ 0.03 to 0.08% among those with *F <* 0.0009 (*SI Appendix*, Table S3).

The phenotypes of the 66 probands with RGs in known human recessive CHD genes were compared to the previously reported phenotypes resulting from RGs in each gene. Among 64 with available CHD phenotype description, 60 probands (94%) had phenotypes that were either concordant or overlapped with prior phenotype descriptions (*SI Appendix*, Table S4 and Dataset S2) supporting the functional relevance of most of these RGs.

RGs in known CHD genes contributed significantly to all phenotypic subgroups included in the cohort except ASD (*SI Appendix*, Table S5). RGs are nearly 10-fold enriched and can explain ~3.4% of HTX in the cohort. RGs account for nearly 2% of nontrisomy 21 atrioventricular canals (AVCs) with RGs accounting for ~1% of each of the remaining phenotypes. Similarly, the broader spectrum of probands with laterality-associated defects including HTX and non-HTX probands with cardiac defects that are frequently observed in HTX also had high enrichment in RGs (enrichment = 7.4, *P* = 5.5 × 10^−16^). Nearly half of the RGs in probands with laterality-associated defects were in genes encoding known ciliary proteins (13 RGs in ciliary genes among 28 RGs in laterality-associated defect cases, enrichment = 6.3, *P* = 2.5 × 10^−7^; *SI Appendix*, Table S6). RGs in non-laterality-associated phenotypes are less enriched and explain smaller fractions of these phenotypes (*SI Appendix*, Table S6).

The known CHD genes with the highest number of RGs were *GDF1* (n = 13); *MYH6* (n = 12); *DNAH5* (n = 7); *PLD1* (n = 6); *DNAH9* and *DNAI2* with three each; *ATIC*, *DNAH11*, *DNYCH1*, *NADSYN1,* and *PKD1L1* with two each; and 12 genes with one RG. The current dataset added two more probands with *GDF1* RGs and six with *MYH6* RGs compared to our previous report (19) (*SI Appendix*, Table S7). It is noteworthy that 54 of the RGs in known CHD genes (82% of the total) came from just 11 genes, each with 2-13 RGs, with another 12 known genes contributing one RG. The remaining 85 known CHD genes had zero RGs in this cohort.

We found three probands with RGs in *NADSYN1 or KYNU*, which encode enzymes in de novo NAD biosynthesis and have been implicated in HLHS or malformation of the aorta and pulmonary artery (30, 31). These probands all had HLHS-related phenotypes. Another proband with HLHS carried an RG in *KMO,* a gene in the same pathway that has not been previously implicated in CHD (*SI Appendix*, Table S8).

### Genome-Wide Analysis of RGs.

**Fig. 1.**
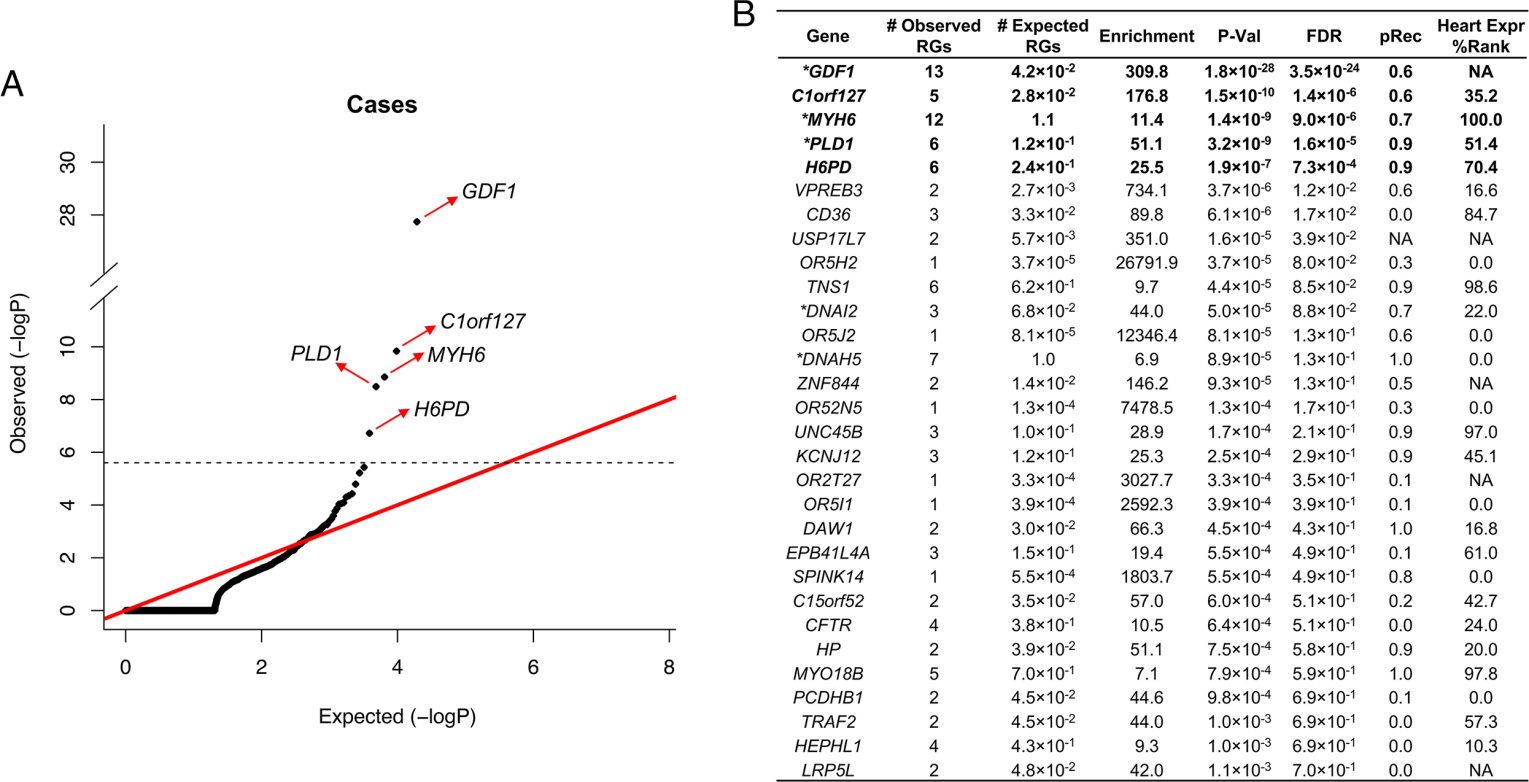
Enrichment of damaging RGs in cases and controls. (*A*) Q-Q plot comparing observed versus expected *P* values for damaging RGs in probands; genes with genome-wide significant enrichment of RGs are marked with a red arrow. The dashed line indicates the genome-wide significant p-value threshold of 2.6 × 10^−6^ (0.05/19,347). (*B*) Top 30 genes from the binomial test. Genes with genome-wide significant burden of RGs are in bold font; * denotes previously identified human recessive CHD genes. pRec: probability of being intolerant to biallelic LoF variants based on gnomAD dataset (doi: 10.1038/nature19057). Heart Expr % Rank: the percentile of gene expression in the mouse heart at embryonic day E14.5 (doi: 10.1038/nature12141).

We next performed a genome-wide analysis for significant recessive risk genes using a binomial test as described above. We identified five genes with genome-wide significant enrichment of damaging RGs (*P* < 2.6 × 10^−6^; Fig. 1), compared with zero genes with significant enrichment in the control cohort (*SI Appendix*, Fig. S2). These genes included two known human recessive CHD genes that we previously reported (*GDF1*, *MYH6*) (19) and three additional genes with genome-wide significance (*C1orf127*, *PLD1,* and *H6PD*) (Fig. 1).

### Recessive *C1orf127* Genotypes Cause Laterality Defects.

**Fig. 2.**
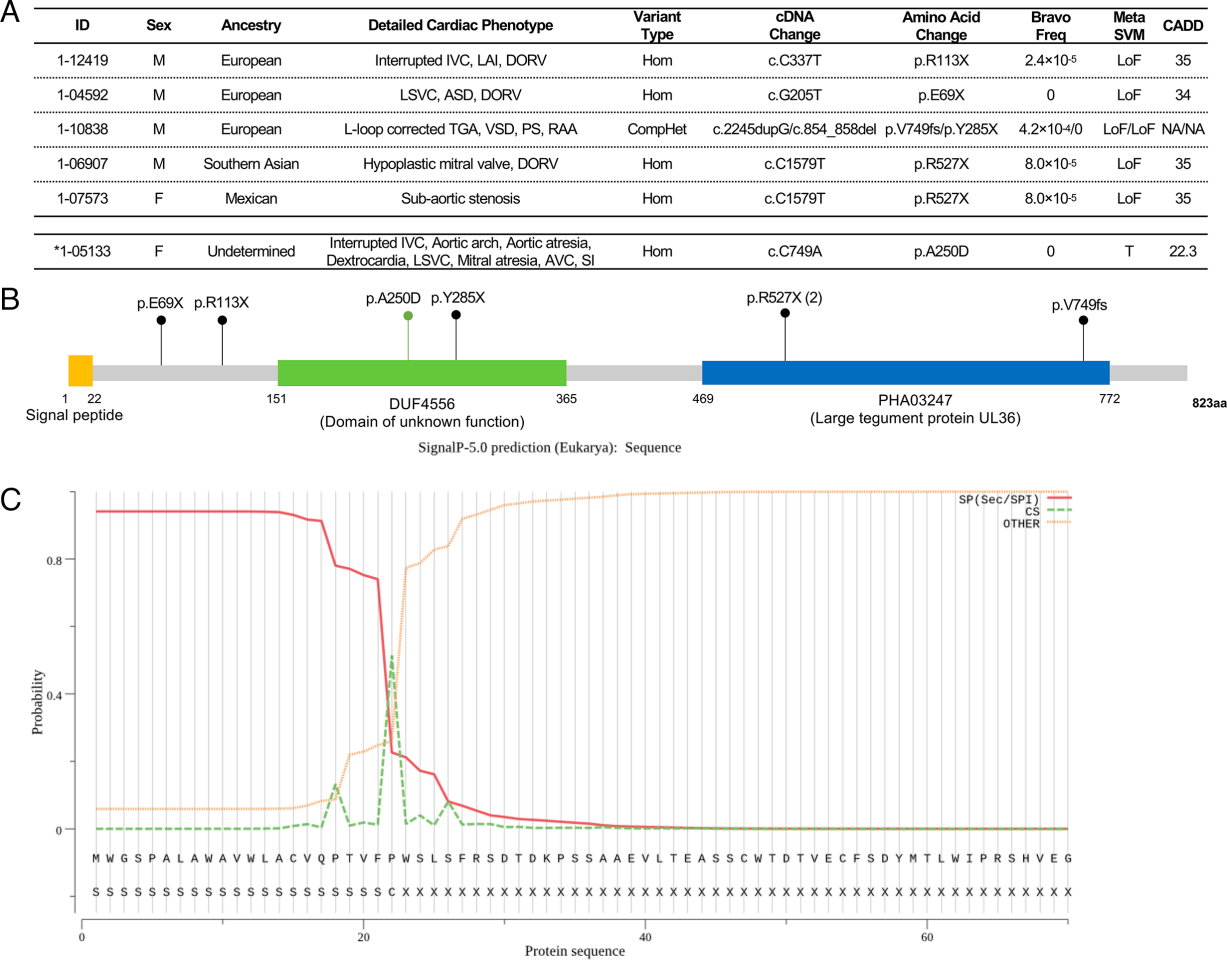
RGs in *C1orf127*, a likely secreted protein. (*A*) RGs in probands with RGs in *C1orf127* and associated phenotypes. HTX: heterotaxy; LVO: left ventricular outflow tract obstruction; CTD: conotruncal defect; IVC: inferior vena cava; LAI: left atrial isomerism; DORV: double-outlet right ventricle; LSVC: Persistent left superior vena cava; ASD: atrial septal defect; TGA: transposition of the great arteries; VSD: ventricular septal defect; PS: pulmonary stenosis; RAA: right aortic arch; AVC: atrioventricular canal; SI: situs inversus. * (below the dashed line), proband identified post hoc with heterotaxy and rare RG predicted as benign by MetaSVM but damaging by SIFT and Polyphen with CADD score > 20. (*B*) Location of *C1orf127* variants on the C1ORF127 protein. C1ORF127 protein contains a likely N-terminal signal peptide, a DUF4556 domain, and a PHA03237 domain. The LoF variants are shown in black while missense variant is shown in green. (*C*) Signal peptide prediction in C1ORF127. The signal peptide was predicted using SignalP-5.0 (http://www.cbs.dtu.dk/services/SignalP/). The red line indicates the probability the sequence comprises a secretory signal peptide (Sec/SPI); the green line indicates the probability of a proteolytic cleavage site; the orange line shows the probability that the sequence does not encode a signal peptide. At the *Bottom* of the figure, the encoded amino-terminal amino acid sequence of C1ORF127 is shown. The cleavage site is predicted to be between amino acids 22 and 23, and is more than twice as likely as the next most likely site. The overall likelihood of the protein having a Sec signal peptide is calculated to be 0.9.

Five unrelated probands had LoF RGs in *C1orf127*, including five different homozygous or compound heterozygous LoF genotypes that were distributed across the encoded protein. These included two homozygotes for p.Arg527X, one each for p.Arg113X (reported in ClinVar 1335917 as VUS in a patient with Heterotaxy) and p.Glu69X, and one compound heterozygote (p.Val749GlyfsTer30/p.Tyr285X) (Fig. 2*A* and *SI Appendix*, Fig. S3). Three of these five probands are from consanguineous union. From the genome-wide binomial test, *C1orf127* showed highly significant enrichment for damaging RGs (enrichment = 176.8, *P* = 1.5 × 10^−10^; Fig. 1*A*) and a greater enrichment for LoF RGs (enrichment = 392.4, *P* = 2.6 × 10^−12^; *SI Appendix*, Fig. S4). There is one prior case report of a damaging recessive genotype in *C1orf127* in related members of a consanguineous kindred who had heterotaxy and complex CHD (32).

Four probands with LoF RGs in *C1orf127* presented with laterality-associated cardiac defects, including DORV and HTX (with dextrocardia, L-TGA, and left atrial isomerism). Other cardiovascular phenotypes associated with laterality defects, including interrupted inferior vena cava and right aortic arch, were also observed. One proband homozygous for the p.Arg527X variant did not have an apparent laterality phenotype, instead having subaortic stenosis. *C1orf127* was thus particularly enriched for LoF RGs among probands with laterality-associated cardiac defects in the cohort (enrichment = 957.8, *P* = 1.1 × 10^−11^, *SI Appendix*, Fig. S5). No extracardiac phenotypes (other than organ laterality defects) were reported in these patients.

*C1orf127* (Chromosome 1 Open Reading Frame 127) encodes a protein with a highly hydrophobic leader sequence in its amino-terminal 22 amino acids, a characteristic of the signal sequence of secreted proteins, along with a strongly predicted protease cleavage site (Fig. 2 *B* and *C*). The *C1orf127* mouse ortholog (*Gm572*) is expressed in the cardiovascular system throughout mouse embryonic development from E9 to P4 (Mouse Genome Informatics), consistent with a role in cardiac development. During mouse gastrulation, *Gm572* is predominantly expressed in notochord (*SI Appendix*, Fig. S6) (33). *C1orf127* is part of a module of genes expressed only in species including humans, mice, *Xenopus,* and fish that depend on motile cilia at the left–right-organizer that is involved in left–right patterning (34). Coupled with the predominant laterality phenotype in these patients, the evidence suggests that C1orf127 is a secreted protein that impacts left–right patterning of the heart.

### Recessive *PLD1* Genotypes Cause Right-Sided Cardiac Valve Defects.

We identified six probands with RGs in *PLD1* (Phospholipase D1) with genome-wide significant enrichment (*P* = 3.2 × 10^−9^, enrichment = 51.1; Fig. 1 and *SI Appendix*, Fig. S7 and Table S9). All probands presented with right heart defects involving various combinations of tricuspid valve abnormalities, pulmonary atresia, right ventricular hypoplasia, and Ebstein anomaly, a severe form of tricuspid valve malformation characterized by the downward displacement of the tricuspid orifice into the right ventricular cavity (35) (*SI Appendix*, Table S9). Variants in *PLD1* (36, 37) have been reported in isolated patients with CHD including right-sided malformations, but this study added genome-wide significant burden through an unbiased analysis of a human CHD cohort. Among 330 probands in our cohort with right-sided valvular defects (defined as tricuspid valve defect, Ebstein anomaly, or pulmonary atresia in the absence of VSD, TOF, HTX, and D-TGA) the enrichment of RGs in *PLD1* was 696.9 fold (*P* = 5.1 × 10^−16^, *SI Appendix*, Fig. S8).

Notably, three of six probands with damaging RGs in *PLD1* had the identical homozygous variant p.Ile668Phe, and all three were of Ashkenazi Jewish (AJ) ancestry (*SI Appendix*, Fig. S9), suggesting a founder variant. Among 410 PCA-defined Ashkenazim in our cohort, the overall allele frequency of p.Ile668Phe was 2.1%, predicting a homozygote frequency of 1 in 2,500. The finding of three homozygotes and 11 heterozygotes among 410 participants strongly violates Hardy–Weinberg equilibrium (*P* = 1.4 × 10^−8^, 1-df χ^2^ test with Yate’s correction), consistent with selection for homozygous *PLD1* genotypes among probands with right heart valvular defects. The maximum shared haplotype bearing the p.Ile668Phe variant among the three homozygous individuals is ~657 kb with an estimated age of ~52 generations (95% CI: 42.4 to 69.0 generations) (*SI Appendix*, Fig. S10). The homozygous p.Ile668Phe genotype accounts for ~12% (3/25) of right-sided valvular defects among Ashkenazim in our cohort.

### Variants in *H6PD*.

The gene *H6PD,* encoding hexose 6-phosphate dehydrogenase, had six RGs, all from rare D-mis variants and surpassing thresholds for genome-wide significance (Fig. 1). This enzyme is responsible for regenerating NADPH, a key cofactor for many anabolic reactions. RGs in *H6PD*, typically featuring LoF variants, have previously been found in patients with cortisone reductase deficiency, which results in cortisol deficiency due to inability to reduce cortisone to cortisol; however, CHD was not reported in any of these people (38). Interestingly, the missense variants show evidence of clustering within the protein, with all probands having two variants that lie between codons 321 and 518. One variant occurred three times in RGs in people of diverse ancestries, indicating it is not a founder variant, but consistent with being selected for its association with CHD. Nonetheless, in contrast with most bona fide CHD genes in which there is some consistency in the phenotypes resulting from variants, probands with *H6PD* RGs had widely varying phenotypes; aside from three having VSDs (and other CHD features), little was shared, with phenotypes including HTX, HLHS, aortic coarctation, single ventricle, and AVC; one patient had hypospadias and cryptorchidism (*SI Appendix*, Table S10). This inconsistency leaves open the possibility that the observed result is a chance event; further results will be required to assess the significance of the observed findings.

### Genes with Low FDR Values.

In addition to genes with RG burdens that surpass thresholds for genome-wide significance, several additional genes not previously implicated in CHD approached this value and had false discovery rate values < 0.05 (*VPREB3, CD36, USP17L7*) or < 0.1 (*OR5H2*, and *TNS1*) (Fig. 1*B*) suggesting that some may prove to be bona fide CHD genes. Among these, *TNS1*, a gene that is highly expressed in the developing heart and has a probability of being intolerant to homozygous LoF variants (pRec) of 0.9 (Fig. 1*B*), encodes tensin, a focal adhesion protein that interacts with actin; *TNS1* had six RGs and lies near a common variant associated with mitral valve prolapse; *Tns1*^−/−^mice and zebrafish have mitral valve defects (39). Five of the probands with RGs in *TNS1* had valve abnormalities affecting the mitral, pulmonary, or aortic valve as part of their CHD, including three with tetralogy of Fallot (*SI Appendix*, Table S11).

### Genes with More than One RG.

Genes with two or more probands with damaging RG are more likely to confer CHD risk. We identified 152 genes with at least two damaging RGs (*SI Appendix*, Table S12), compared with 117.8 genes expected by permutation analysis (*P* = 2.1 × 10^−5^, enrichment = 1.3, *SI Appendix*, Table S13). In contrast, no enrichment was observed for genes with 2 or more synonymous RGs (enrichment = 1.0, *P* = 0.6; *SI Appendix*, Table S13). Of these 152 genes, 11 were known CHD genes, leaving 141 candidate CHD genes with two or more RGs vs. 106.5 expected (*P* = 1.9 × 10^−5^, enrichment = 1.3 [1.2, 1.6], *SI Appendix*, Table S13) suggesting that ~35 genes in this set may be bona fide CHD genes that may be validated in larger cohorts. The 152 genes with more than one damaging RG are significantly more connected than expected by STRING analysis (398 observed edges compared to 85 expected edges, *P* < 1.0 × 10^−16^) and identify subclusters including cilia and cardiomyocyte/sarcomere proteins (40) (*SI Appendix*, Fig. S11).

### Clustering of RGs in Cardiomyocyte and Notochord Lineages.

**Fig. 3.**
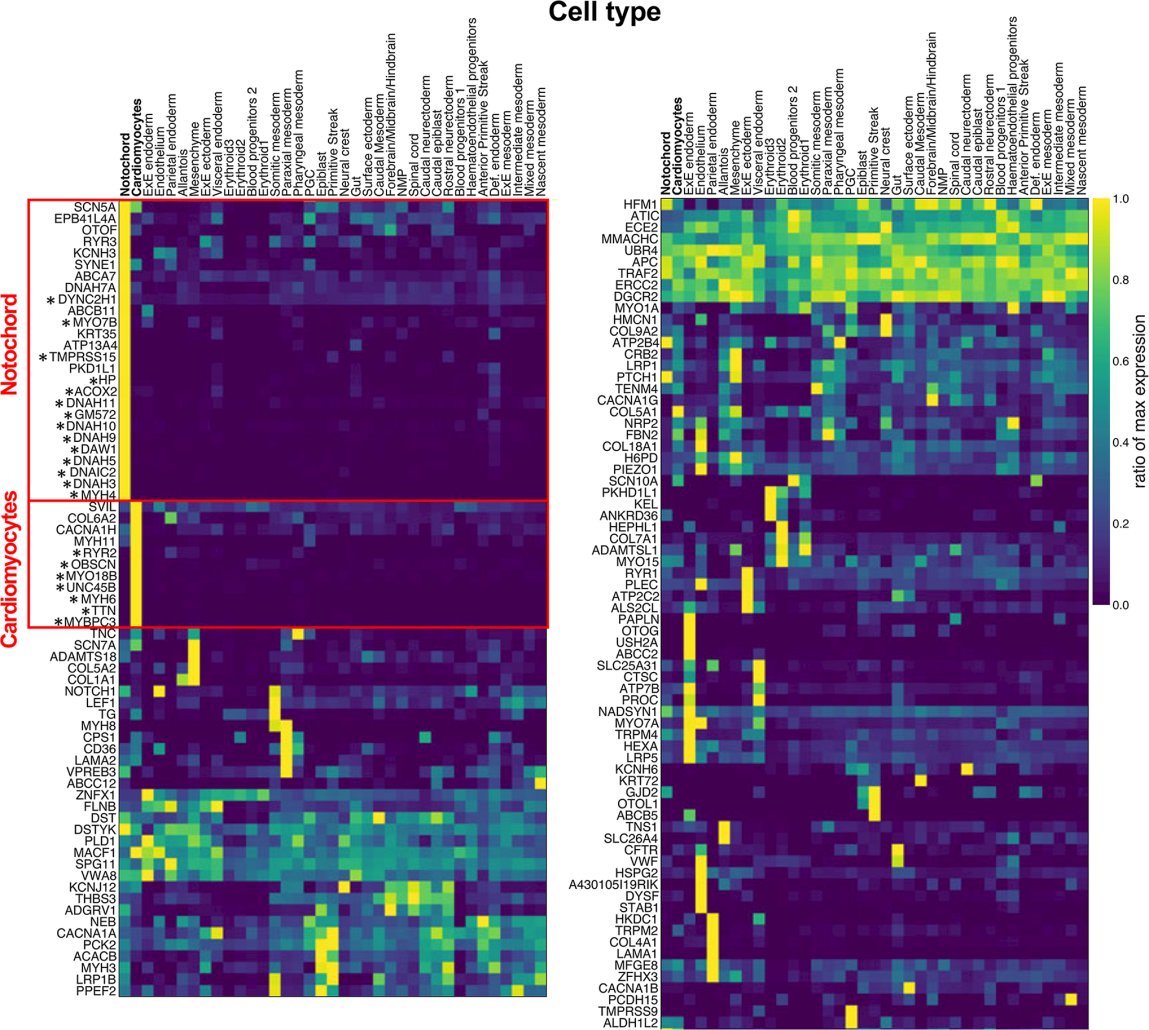
Single-cell expression of genes with ≥ 2 RGs during mouse gastrulation. The single-cell RNA-seq data of mouse gastrulation were acquired from Pijuan-Sala et al(33). The expression of each gene was normalized to the level of its expression in the cell/tissue type with highest expression (ratio of max expression). Genes were then clustered by their ratio of max expression in different cell/tissue types using the UPGMA hierarchy clustering algorithm. Only genes with available expression data were shown. *indicates genes with at least fivefold higher expression in notochord or cardiomyocytes compared to other tissues. The *x*-axis indicates the cell types, the *y*-axis shows the genes with ≥ 2 damaging RGs. The column on the *Right* is a continuation of the column on the *Left*.

Cardiac morphogenesis occurs early in development, between days 15 to 56 postconception in humans and embryonic days 7.5 to 14.5 in the mouse. To better understand the relationship between recessive genes and CHD phenotypes, we examined the expression of genes harboring two or more RGs across different cell types during mouse gastrulation using a single-cell RNA-sequencing dataset collected at embryonic days 6.8 to 8.5 (33). 141/152 of these genes have available expression information in this dataset. To identify genes with tissue-specific function that may contribute to a specific cardiac phenotype, we clustered the genes using their ratio of maximum expression across all the tissues studied. We defined a gene as showing specific expression if its expression is ≥ fivefold higher in one tissue compared to other tissues studied. Two major clusters of genes with tissue-specific expression were observed: one group with predominant notochord expression and another with predominant cardiomyocyte expression (boxed in red in Fig. 3).

There are fourteen genes with notochord-specific expression, including mouse orthologs of six recessive human CHD genes that collectively have 22 RGs in the cohort, these include *C1orf127* (5 RGs) and five known human CHD genes *DNAH5* (7 RGs), *DNAH9* (3 RGs), *DNAH11* (2 RGs), *DYNC2H1* (2 RGs), *DNAI2* (3 RGs). In addition, the mouse ortholog of the human gene *DAW1*, is a known mouse CHD gene and has two RGs. There were 24 RGs among these seven genes. Notochord-expressed genes include genes required for cilia structure and function, and notochord cells include cells derived from the ciliated embryonic left–right organizer (41). Variants affecting cilia and left–right organizer structure and function are associated with a wide range of CHD with and without organ heterotaxy (42, 43). This is reflected in our finding that the 24 probands with RGs in these seven genes presented with a wide range of CHDs including 13/24 who had laterality-associated defects.

Similarly, there are seven cardiomyocyte-specific expressed genes with at least two RGs, including the known recessive CHD gene *MYH6* (19). Cardiomyocytes are the contractile cells of the heart and their dysfunction is associated with cardiomyopathy. It is hypothesized that cardiomyocyte dysfunction may lead to structural heart defects, especially those on the left side of the heart (19, 44, 45). We examined the enrichment of RGs in all cardiomyocyte-specific genes in left-sided CHD probands. We observed a 1.8-fold of enrichment of RGs in these genes among 1,849 cases with a left-sided defect (*P* = 1.2 × 10^−3^; *SI Appendix*, Table S14), including seven genes with two or more RGs. No enrichment was observed in 3,445 cases without left-sided defect (*P* = 0.3, enrichment = 1.1) or in controls (*P* = 0.5, enrichment = 1.0; *SI Appendix*, Table S14).

**Fig. 4.**
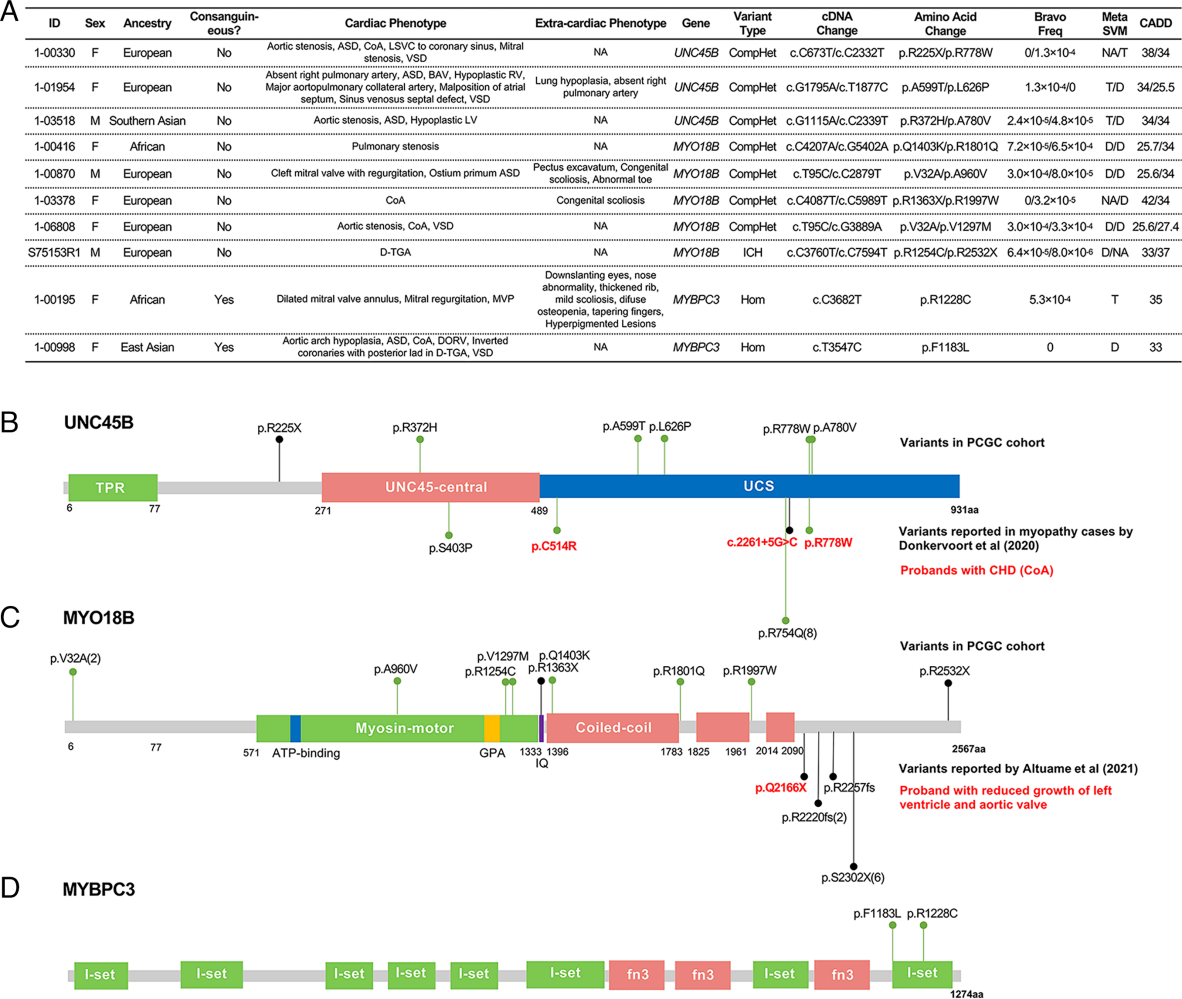
RGs and phenotypes in *UNC45B*, *MYO18B*, and *MYBPC3*. (*A*) RGs in *UNC45B*, *MYO18B*, and *MYBPC3* in probands with CHD. ASD; CoA: coarctation; LSVC: left superior vena cava; VSD: ventricular septal defect; BAV: bicuspid aortic valve; RV: right ventricle; LV: left ventricle; MVP: mitral valve prolapse; DORV; D-TGA: D-transposition of great artery. (*B*–*D*) Location of variants in UNC45B (*B*), MYO18B (*C*), and MYBPC3 (*D*). Variants denoted above each gene were found in probands with CHD described in (*A*) while variants below each gene are previously reported in patients with RGs and cardiomyopathy and/or CHD. LoF variants are shown in black and missense variants are shown in green. Numbers in parentheses indicate the number of probands with the variant. (*B*) UNC45B contains the N-terminal TPR domain which interacts with Hsp90, a conserved central domain, and a C-terminal UCS domain which binds myosin. Variants reported in ref. 48 are shown. Variants found in patients with CoA in both cohorts are labeled in red. (*C*) MYO18B contains an N-terminal myosin-motor domain, a myosin light chain binding IQ motif, and 3 C-terminal coiled-coil regions which might allow for dimerization. Variants found in our cohort are mostly missense and distribute across the encoded protein, RGs reported by Altuame et al (49) in patients with Klippel–Feil syndrome with cardiomyopathy are all LoF and cluster in the C-terminal end of the protein. One patient with p.Gln2166X variant reported reduced growth of the left ventricle and aortic valve. (*D*) MYBPC3 contains eight Ig-like C2 (I-set) domains and three fibronectin type-III (fn3) domains. Both of the variants in our cohort are missense and map to the last I-set domain, which is known to bind to the myosin rod (53).

In addition to *MYH6*, three of the seven cardiomyocyte-specific genes with two or more RGs showed at least fivefold enrichment of damaging RGs compared to expectation at the gene-level: *UNC45B*, *MYO18B,* and *MYBPC3*. Like *MYH6,* all three have previously been implicated in cardiomyopathy (46–50). Three damaging compound-heterozygous RGs were identified in *UNC45B* (Fig. 4 *A* and *B*), representing a 29-fold enrichment over expectation (*P* = 1.7 × 10^−4^). *UNC45B* encodes a cochaperone protein that plays a crucial role in stabilization of cardiac myosin and organization of myofibrillar heart contractility (51). *UNC45B* is associated with myofibrillar myopathy through recessive inheritance (OMIM# 619178). Mice with recessive LoF in *Unc45b* arrest cardiac morphogenesis at E9.5 and have failure of contractile function (52). All three probands with *UNC45B* RGs had left-sided heart defects that included aortic stenosis or bicuspid aortic valve as part of complex CHD. Proband 1-00330 carried a compound heterozygote with one p.Arg778Trp variant previously reported in a patient with coarctation of the aorta (CoA) (48), which was part of the CHD in proband 1-00330.

Five patients had compound-heterozygous RGs in the myosin gene *MYO18B* (Fig. 4 *A* and *C*). *MYO18B* is associated with the recessive Klippel–Feil syndrome (OMIM# 616549), characterized by myopathy and facial dysmorphism. While variants are seen across the length of the encoded protein, there is some clustering near the end of the motor domain and the beginning of the coiled-coil domain. Three of five probands showed left-sided heart defects. Two others reported congenital scoliosis, which has been found in approximately half of recessive Klippel–Feil cases with *MYO18B* variants (49). Zebrafish and mouse models null for *MYO18B* showed cardiac dysfunction and embryonic lethality (54, 55). Human *MYO18B* biallelic LoF variants have been associated with cardiomyopathy, and reduced growth of the left ventricle and aortic valve (56); in addition, the MYO18B locus was implicated in a GWAS analysis of patients with HLHS (45).

Last, two probands had homozygous *MYBPC3* (myosin-binding protein C3) missense variants (Fig. 4 *A* and *D*). Both variants lie in the last immunoglobulin I-set domain of the protein (Fig. 4*D*), which is known to bind to the myosin rod (53). One proband had a dilated mitral valve annulus, mitral regurgitation, and MVP. The other had D-TGA with CoA. *MYBPC3* is the cardiac isoform of myosin-binding protein C and is predominantly expressed in cardiomyocytes. Monoallelic *MYBPC3* LoF variants are the most common genetic cause of autosomal dominant hypertrophic cardiomyopathy, and biallelic truncating variants cause severe neonatal cardiomyopathy, while monoallelic missense variants are associated with dilated and hypertrophic cardiomyopathy (57, 58) (OMIM# 615396 and 115197).

### Estimation of the Recessive Contribution to CHD.

To estimate the overall contribution of RGs to CHD, we adopted the method of Martin et al. (59), which assumes that parents are relatively depleted of damaging RGs that cause severe disease, while probands are enriched. The relative enrichment of RGs in probands compared to their parents reflects the percentage of probands in whom RGs contribute to CHD risk (*Materials and Methods*). From the analysis of all 3,716 trios, we estimate that damaging RGs contribute to disease risk for 2.2% probands (CI: 0.8 to 3.5%; Table 2). Across the entire cohort of 5,424 probands, RGs in previously known recessive human CHD genes plus those implicated herein can account for 1.3% of all probands. Thus, RGs in known CHD genes can account for 62% of the contributing RGs, indicating that many recessive CHD genes likely remain to be discovered.

**Table 2.** Estimation of the contribution of RGs in CHD trios

Type of RGs	# Expected RGs in 7,432 Parents	# Observed RGs in 7,432 Parents	# Observed / # Expected in Parents	# Expected RGs in 3,716 Probands	# Observed RGs in 3,716 Probands	# Observed / # Expected in Probands	Fraction of Probands with Causal RGs	CI
Syn	4122.2	4,135	1.0	1951.5	1951	1.0	-	-
LoF	156.5	95	0.6	69.9	61	0.9	0.5%	[0.1%,0.9%]
Damaging	1418.0	1,181	0.8	659.1	630	1.0	2.2%	[0.8%,3.5%]

Syn: synonymous RGs; LoF: loss-of-function RGs; Damaging: All damaging RGs, including LoF and D-mis.

We also estimated the contribution of RGs to CHD probands from consanguineous union; this was estimated to be 6.9% (CI: −0.2% to 14.0%) compared to 1.6% (CI: 0.4% to 2.7%) in probands from nonconsanguineous unions (*SI Appendix*, Table S15). Including the three *C1orf127* RGs among consanguineous probands, we identified RGs in known genes in 35/675 consanguineous probands (5.2%), inferring that variants in identified recessive CHD genes account for ~75% of all RGs in this cohort. Similarly, including two nonconsanguineous probands with RGs in C1orf127, RGs explained 0.8% (36/4749) of this group.

We then estimated the overall contribution of RGs to CHD phenotypic subsets. RGs were estimated to contribute to 5.4% (CI: 2.1% to 8.6%) of probands with laterality-associated cardiac defects compared to 1.4% (CI: −0.05% to 2.8%) of those without laterality-associated defects (*SI Appendix*, Table S16). This is consistent with our previously published data (19). Consistent with these estimates, we identified RGs in known genes in 2.4% of probands with laterality-associated defects, and 0.9% of the remainder.

Due to the contribution of founder variants in the AJ population, we also separately estimated the fraction of damaging RGs that contribute to AJ probands. From PCA of common variants from ES, there were 279 trios and 131 singletons inferred to be of AJ descent (*SI Appendix*, Fig. S9). From the trios, we estimated that damaging RGs collectively contribute to 4.6% of CHD in this population (CI: −0.7% to 9.8%) (*SI Appendix*, Table S17). Thus, because the two identified founder variants in AJ (*GDF1* p.Met364Thr, and *PLD1* p.Ile668Phe) account for recessive CHD in 14/410 probands (3.4%), these founders account for an estimated 74% of the recessive signal in AJs in this cohort.

## Discussion

Our study has characterized the spectrum and frequency of RGs contributing to CHD in the largest cohort reported to date, providing the opportunity to determine the frequency and phenotypic spectrum resulting from biallelic variants in known recessive CHD genes in probands ascertained without bias for particular CHD phenotypes, as well as the identification of additional CHD genes.

Among the 5,424 probands, RGs in the 108 previously known recessive CHD genes were only found in 66 probands (1.2%, increasing to 1.3% including the five RGs in gene *C1orf127*), and only 23 known recessive CHD genes had even a single RG. RGs in four genes occurred in 6 to 13 probands each and accounted for 58% of the total; two of these were driven by founder variants among Ashkenazim (*SI Appendix*, Table S4). These results indicate the rarity with which RGs in most of these CHD genes contribute to disease in this cohort ascertained from tertiary referral centers.

Importantly, despite comprising only 12.4% of the cohort, probands from consanguineous union accounted for more than half of the RGs in CHD genes (51.5%). 4.8% of probands from consanguineous union had RGs in known genes compared to only 0.71% of probands from nonconsanguineous union (6.7-fold enrichment). The percentage of probands explained by homozygous variant in known CHD genes increased with increased levels of consanguinity: 0.08% in the absence of consanguinity; 1.3% among likely 4th cousin unions; 4.5% for 3rd cousins, 3.9% for 2nd cousins, and 9.5% for 1st cousins or closer (*SI Appendix*, Table S3).

An independent statistical test of the impact of RGs across the entire cohort (59) supports the relative rarity of recessive contribution to CHD, concluding that RGs contribute to ~2.2% of probands overall (~117 probands). Because RGs in known genes can account for 1.3% of all probands, these RGs can account for ~61% of the total number of RGs contributing to CHD in the cohort, leaving ~0.8%, or ~45 probands in the cohort with RGs in genes that are not yet clearly identified as CHD genes. Among these, orthologs of mouse CHD genes are excellent candidates, including *TNS1* and *DAW1*, which had six and two RGs, respectively, and CHD phenotypes related to those found in the mouse. Similarly, after excluding known human recessive CHD genes, there are 437 RGs in genes previously associated with human phenotypes other than CHD (enrichment = 1.0, *P* = 0.9). Nonetheless, the rarity of RGs in so many known genes is striking and suggests population frequency < 1/500,000 (assuming overall rate of severe CHD of ~0.01). This could be explained by pre- or perinatal lethality for many of the RGs in these genes. The total number of additional recessive CHD genes that have not yet been discovered is likely to be large.

The estimated fraction of cases accounted for by RGs in probands with laterality-associated phenotypes (~5.4%) indicates that this subgroup is an outlier, compared to a frequency of only 1.4% of the remainder. The empirical data support this estimate. RGs in CHD genes known to cause laterality – associated phenotypes account for 3.7% (n = 43) of the 1,160 probands with these type of malformations, and 61% of the total set of RGs in known CHD genes.

This cohort demonstrates the challenge inherent in identifying recessive CHD genes from large cohort studies from the general population. The complete list of damaging RGs found in this cohort and their associated phenotypes (Dataset S2) will help identify additional recessive CHD genes. The marked enrichment in discovery of RGs in probands from consanguineous union, as well as the discovery of founder variants in populations that have gone through recent bottlenecks, makes clear that prioritizing recruitment of such probands will maximize the opportunity for recessive CHD gene discovery. In contrast to the relatively small contribution to CHD by biallelic variants, monoallelic de novo mutations account for a much larger fraction of CHD, ~9%.

One limitation of this study is that tools to distinguish damaging from benign missense variants have incomplete sensitivity and specificity. When damaging RGs are uncommon in a study population, stringent criteria help prevent loss of power but reduce sensitivity. Nonetheless, once a gene has been well implicated using stringent criteria, application of the same criteria may be inappropriately insensitive in clinical practice, where the posterior probability of a proband by chance having a specific rare phenotype and an RG in a gene known to produce that phenotype but which has higher allele frequency than the threshold used for gene discovery. For example, in our dataset, we found three probands had RGs in *NADSYN1* due to the same variant that barely exceeded the MAF cutoff of 10^−3^ in gnomAD, with MAF of 1.2 × 10^−3^. All three patients had mitral valve abnormalities, consistent with characteristics of phenotypes seen in other patients with RGs in *NADSYN1* (*SI Appendix*, Table S8). Similarly, one patient had a potentially clinically significant RG in *C1orf127* (p.Ala250Asp, MetaSVM “T”) that was predicted damaging by SIFT (60) and Polyphen (61). This patient had a HTX phenotype that included left atrial isomerism (Fig. 2*A* and *SI Appendix*, Fig. S3) consistent with phenotypes found in other *C1orf127* patients. Further, two probands had rare homozygous genotypes in *PLD1*: p.Tyr894Ser (MetaSVM T and CADD = 28) and p.Tyr818His (MetaSVM T and CADD = 23) (*SI Appendix*, Fig. S7 and Table S9) both of whom presented with right-sided valvular defects. These subjects were not included in the summary data but highlight the distinction between the goals of disease gene discovery and clinical application. Addition of subjects such as these to the total could also reduce the gap between anticipated and observed RGs in a cohort.

Differences between cases and controls in this study also introduce potential limitations. The small differences in ethnic distribution and sequencing metrics (*SI Appendix*, Tables S1 and S2) have no impact on the gene discovery analysis, which used a binomial test that is fully independent of the control cohort. Comparison of the results of this test showed that the binomial test for RGs in controls showed no departure from expectation in Q-Q plots. Further, RGs in known recessive CHD genes were 11-fold more frequent in cases than controls, providing confidence that sequencing and genotyping errors were not generating large numbers of false positive results. Parental consanguinity was significantly higher in CHD probands despite ascertainment of cases based solely on presence of CHD; parental consanguinity is expected to be enriched among kindreds segregating recessive traits, but not in others. The lower frequency of parental consanguinity in autism kindreds suggests a lower contribution from RGs, supported by a recent well-designed study from Qatar, where consanguinity is prevalent, parental consanguinity was not increased in autistic compared to nonautistic probands (62).

By mapping genes with RGs in CHD patients to mouse embryo single-cell RNAseq data, we showed that the embryonic expression pattern of a CHD gene impacts the final CHD phenotype. Previous studies found CHD-causing RGs clustering in genes associated with cilia function that were especially enriched in laterality-associated CHD. Our analysis confirmed those findings by identifying RGs enriched in genes expressed in the embryonic notochord, which is developmentally related to the ciliated embryonic left–right organizer. The notochord-expressed genes include a genome-wide significant CHD gene, *C1orf127*, encoding a likely secreted protein with a role in the development of left–right asymmetry. Importantly, in addition to cilia-associated genes, we identified recessive variants in four genes previously implicated in cardiomyopathy (*MYH6*, *UNC45B*, *MYO18B*, and *MYBPC3*) that are expressed in embryonic cardiomyocytes; RGs in these genes contributed to left-sided CHD phenotypes. We propose that the dysfunctional cardiomyocytes resulting from RGs may impact ventricular contraction and embryonic blood flow, leading to structural CHD such as an underdeveloped left ventricle and/or malformed aortic and mitral valves, since there is emerging evidence for interdependence between embryonic hemodynamics and cardiac morphogenesis (44). These findings also point to a population of CHD patients who may be at increased lifetime risk of myocardial dysfunction and heart failure.

The overall contribution of rare mutations with large effect to severe CHD is at least 35%. This derives from ~15% of CHD attributable to large damaging de novo copy number variants; another 10% having chromosomal aneuploidy (trisomy 21, 13, or 18) (10); an excess burden of 8.3% of probands having damaging monoallelic de novo variants compared to controls (19), and 2.2% with biallelic variants as reported herein. It is likely that transmitted monoallelic variants will also play a significant role as larger studies are performed; for example, frequent transmission with incomplete penetrance of LoF variants in *FLT4* has been demonstrated (19).

The etiology of the remaining ~65% of probands remains unknown, though significant risk factors have been identified. In two national cohort studies that each included more than 2 million live births, pregestational maternal diabetes increased the risk of CHD 4-fold across a broad range of CHD subtypes (increasing absolute risk of CHD by ~2.4%) (63, 64). One of these (64) also found odds ratios > 3 for multifetal pregnancy, maternal CHD, and maternal connective tissue disease, along with smaller effects from maternal hypertension, alcohol use, increased maternal age, and others. Collectively, this study suggested such factors can account for ~14% of CHD (64).

The etiology of the remaining ~50% of CHD can include variants in noncoding DNA (20, 65), DNMs predicted to affect gene transcription were enriched 1.15-fold in CHD probands compared to controls, and were found in enhancers of genes implicated in CHD in ~3% of probands, though only 20% of the enhancer variants tested altered gene transcription in vitro (20). Oligogenic models are similarly limited by sample size and potential heterogeneity of interacting loci; nonetheless, an example of digenic contribution to HLHS has been reported in mice (66).

The contribution of common variants with smaller effects has similarly been limited by small sample sizes and the genetic heterogeneity of CHD subtypes. A study of 757 probands with ASD showed significant association at chromosome 4p16, with an odds ratio of 1.4 (67). Similarly, a GWAS of 1145 probands with left-sided CHD identified significant association with a chromosome 20 interval that included *MYH7b and miR499A* with an odds ratio of 1.5 (68). Effects of common variants could also modify penetrance of rare variants. Like for other diseases, the study of much larger cohorts will be needed to provide more robust identification of the contribution of common variants to CHD risk (69).

Stochastic processes are yet another potential contributor to disease pathogenesis. Given that ~60% of the signal from damaging DNMs occurs in genes with high pLI (19), indicating dosage sensitivity, stochastic phenocopies of reduced gene expression of high pLI genes in critical cell types at key times in development could account for significant fractions of CHD.

In summary, results from WES of 5,424 CHD probands have further identified genes and pathways implicated in CHD, have provided estimates of the contribution of recessive inheritance to CHD, and demonstrated the marked enrichment of probands resulting from parental consanguinity and founder variants. These findings can guide the genetic testing of CHD and can help stratify patients into phenotypic groups to support personalized medical management. Future efforts to sequence larger CHD cohorts, particularly probands from consanguineous unions and founder populations, are likely to be the most efficient approach to identify new recessive risk genes and mechanisms for CHD.

## Materials and Methods

### Study Participants.

A total of 3,716 child–parent trios and 1,708 singleton probands were recruited to the CHD Network Study of the PCGC (CHD GENES: ClinicalTrials.gov identifier NCT01196182) (70) as previously described. All participants or their parents provided informed consent using protocols that were reviewed and approved by institutional review boards at participating institutions. A total of 1,798 unaffected siblings of people with autism and their unaffected parents were obtained from the Simons Simplex Collection (SSC) as the controls. The access to the data was acquired from SSC on the National Institute of Mental Health Data Repository. Written informed consent was obtained from all participants as provided by the Simons Foundation Autism Research Initiative. See *SI Appendix*, *Materials and Methods* for details.

### Exome Sequencing and Analysis.

For CHD cases, DNA samples from venous blood or saliva were extracted and sequenced at the Yale Center for Genome Analysis as described before (19). 1,798 sibling-parent trios with WES from Simons Simplex Collection served as controls. See *SI Appendix*, *Materials and Methods* for details.

### Variant Filtering.

Damaging variants were defined as likely LoF variants (“LoF”, comprising stopgain, stoploss, frameshift insertion/deletion, or variants altering canonical splice sites), D-mis variants (“D-Mis”, defined as missense variants predicted as deleterious by MetaSVM and/or having a CADD score ≥ 30), plus nonframeshift indels. Homozygous variants were called from all probands. Compound heterozygous variants were called from trio samples. Variant pairs falling in the same genes in singleton probands were inferred to be compound heterozygotes if they were predicted to be in trans based on the absence of their co-occurrence in individuals in the gnomAD database using the tool (https://gnomad.broadinstitute.org/variant-cooccurrence). See *SI Appendix*, *Materials and Methods* for details.

### Consanguinity Determination.

Beagle v3.3.2 (71) was used to perform haplotype phasing and the calculation of the inbreeding coefficient. Consanguineous participants were defined as having inbreeding coefficient at least 0.0009, which is close to the expectation of the offspring of fourth cousin union. See *SI Appendix*, *Materials and Methods* for details.

### Gene Enrichment Analysis.

The expected number of recessive variants was estimated as described previously (19) by assuming that the expected frequency of damaging recessive variants in each gene would be proportional to the de novo mutability in each gene, followed by a one-tailed binomial test. The Bonferroni multiple testing threshold is 2.6 × 10^−6^ (0.05/19,347) for the gene-based test. The number of genes with more than one RG was estimated using a permutation test as described before (19). See *SI Appendix*, *Materials and Methods* for details.

### Single-Cell RNA-Seq Analysis.

The single-cell RNA-seq data of mouse gastrulation were acquired from Pijuan-Sala et al. (33). The mouse gene names were matched to their human homolog through bioMart (72) and manual inspections. Genes harboring at least two RGs and with available expression data were clustered by their ratio of max expression in different cell types using UPGMA hierarchical clustering algorithm through python package seaborn v0.11.0. A gene is considered as specifically expressed in one tissue if its expression is at least five-fold higher in that tissue compared to any other tissues. See *SI Appendix*, *Materials and Methods* for details.

### Recessive Contribution Estimation.

The contribution of the RGs to the CHD in the cohort was estimated, as previously described (59). For calculation of the in-cohort allele frequency and to avoid overestimation of expected RGs from false-positive variants, only the variants that passed the following criteria were kept: GQ ≥ 20, DP ≥ 8, Mapping Quality score [MQ] ≥ 40, variant allele fraction ≥ 25%, at least three supporting reads, and not in SegDup regions in all three members of a trio. When estimating RG expectation in phenotype subsets, in-cohort allele frequency, LD pruning, and ROH calculation were all based on samples within each subphenotype. See *SI Appendix*, *Materials and Methods* for details.

## Supplementary Material

Appendix 01 (PDF)

Dataset S01 (XLSX)

Dataset S02 (XLSX)

Dataset S03 (XLSX)

## Data Availability

All PCGC phenotype and DNA sequence data have been deposited in NHLBI’s BioData Catalyst https://biodatacatalyst.nhlbi.nih.gov under dbGaP accession number phs001194 and sub-studies (73).
