# Supplementary material for: Recessive genetic contribution to congenital heart disease in 5,424 probands
Source: Proc Natl Acad Sci U S A. 2025 Mar 3;122(10):e2419992122. doi: 10.1073/pnas.2419992122 (PMC11912448; doi:10.1073/pnas.2419992122)
Supplement: Supplementary file 1 — Appendix 01 (PDF) [file pnas.2419992122.sapp.pdf]

PNAS

Supporting Information for

Recessive genetic contribution to congenital heart disease in 5,424 probands.

Weilai Dong[a,b], Sheng Chih Jin[b,c,d], Michael C. Sierant[a,b], Ziyu Lu[e], Boyang Li[f], Qiongshi Lu[g], Sarah U. Morton[h,i,j], Junhui Zhang[a], Francesc López-Giráldez[k], Carol Nelson-Williams[a], James R. Knight[k], Hongyu Zhao[a,f], Junyue Cao[e], Shrikant Mane[k], Peter J. Gruber[l], Monkol Lek[a], Elizabeth Goldmuntz[m], John Deanfield[n], Alessandro Giardini[o], Seema Mital[p], Mark Russell[q], J. William Gaynor[r], James F. Cnota[s], Michael Wagner[t,u], Deepak Srivastava[v], Daniel Bernstein[w], George A. Porter Jr[x], Jane Newburger[y], Amy E. Roberts[y], Mark Yandell[z], H Joseph Yost[z,aa], Martin Tristani-Firouzi[bb], Richard Kim[cc], Jonathan Seidman[dd], Wendy K. Chung[ee,ff,gg], Bruce D. Gelb[hh,ii], Christine E. Seidman[jj,kk], Richard P. Lifton[b,1], Martina Brueckner[a,ll,1]

[a]Department of Genetics, Yale School of Medicine, New Haven, CT 06510
[b]Laboratory of Human Genetics and Genomics, The Rockefeller University, New York, NY 10065
[c]Department of Genetics, Washington University School of Medicine, St. Louis, MO 63110
[d]Department of Pediatrics, Washington University School of Medicine, St. Louis, MO 63110
[e]Laboratory of Single-cell Genomics and Population Dynamics, The Rockefeller University, New York, NY 10065
[f]Department of Biostatistics, Yale School of Public Health, New Haven, CT 06510
[g]Department of Biostatistics & Medical Informatics, University of Wisconsin, Madison, WI 53706
[h]Division of Newborn Medicine, Department of Pediatrics, Boston Children's Hospital, Boston, MA 02115
[i]Manton Center for Orphan Disease Research, Boston Children's Hospital, Boston, MA 02115
[j]Broad Institute of Massachusetts Institute of Technology and Harvard, Boston, MA 02142
[k]Yale Center for Genome Analysis, Yale University, New Haven, CT 06516
[l]Department of Surgery, Yale University School of Medicine, New Haven, CT 06510
[m]Division of Cardiology, Children's Hospital of Philadelphia, Department of Pediatrics, Perelman School of Medicine, University of Pennsylvania, Pennsylvania, PA 19104
[n]Institute of Cardiovascular Science, University College London, London WC1E 6BT, United Kingdom
[o]Pediatric Cardiology, Great Ormond Street Hospital, London WC1N 3JH, United Kingdom
[p]Division of Cardiology, Department of Pediatrics, The Hospital for Sick Children, University of Toronto, Toronto, ON M5G1X8, Canada
[q]Department of Pediatrics and Communicable Diseases, University of Michigan, Ann Arbor, MI 48109
[r]Division of Cardiothoracic Surgery, Children's Hospital of Philadelphia, Philadelphia, PA 19104
[s]Division of Cardiology, Cincinnati Children's Hospital Medical Center, Cincinnati, OH 45229
[t]Division of Biomedical Informatics, Cincinnati Children's Hospital Medical Center, Cincinnati, OH 45229
[u]Division of Biostatistics and Epidemiology, Cincinnati Children's Hospital Medical Center, Cincinnati, OH 45229
[v]Gladstone Institute of Cardiovascular Disease, San Francisco, CA 94158
[w]Department of Pediatrics, Cardiology, Stanford University, Stanford, CA 94304

[x]Department of Pediatrics, The School of Medicine and Dentistry, University of Rochester Medical Center, Rochester, NY 14642
[y]Department of Cardiology, Boston Children's Hospital, Boston, MA 02115
[z]Department of Human Genetics, University of Utah and School of Medicine, Salt Lake City, UT 84112
[aa]The Catholic University of America, Washington, DC 20064
[bb]Division of Pediatric Cardiology, University of Utah, Salt Lake City, UT 84112
[cc]Pediatric Cardiac Surgery, Smidt Heart Institute, Cedars-Sinai Medical Center, Los Angeles, CA 90048
[dd]Department of Genetics, Harvard Medical School, Boston, MA 02115
[ee]Department of Pediatrics, Boston Children's Hospital, Harvard Medical School, Boston, MA 02115
[ff]Department of Pediatrics, Columbia University Medical Center, New York, NY 10032
[gg]Department of Medicine, Columbia University Medical Center, New York, NY 10032
[hh]Mindich Child Health and Development Institute, Icahn School of Medicine at Mount Sinai, New York, NY 10029
[ii]Department of Pediatrics, Icahn School of Medicine at Mount Sinai, New York, NY 10029
[jj]Cardiovascular Division, Brigham and Women's Hospital, Boston, MA 02115
[kk]HHMI, Chevy Chase, MD 20815
[ll]Department of Pediatrics, Section of Cardiology, Yale School of Medicine, New Haven, CT 06510
[1]Corresponding Authors.
Email: rickl@rockefeller.edu (Richard Lifton), martina.brueckner@yale.edu (Martina Brueckner)

This PDF file includes:

Supporting text
Figures S1 to S11
Tables S1 to S16
Legends for Datasets S1 to S3
SI References

Other supporting materials for this manuscript include the following:

Datasets S1 to S3

Supporting Information Text

Materials and Methods.

Cohort recruiting and description

A total of 3,716 child-parent trios and 1,708 singleton probands were recruited to the Congenital Heart Disease Network Study of the Pediatric Cardiac Genomics Consortium (CHD GENES: ClinicalTrials.gov identifier NCT01196182). Written informed consent for genetic studies was obtained from all participants. Only participants with structural CHD (excluding PDA associated with prematurity and pulmonary stenosis associated with twin-twin transfusion) were included. The cardiac diagnoses were based on the review of imaging and operative reports and were entered as Fyler codes based on the International Pediatric and Congenital Cardiac Codes (http://www.ipccc.net/). Samples were excluded if they have one of the following genetic syndromes: Cat Eye, DiGeorge/Velo-Cardio-Facial/Cayler cardiofacial, Jacobsen, trisomy 21, Turner, and Williams syndrome.

A total of 1,798 unaffected siblings of people with autism and their unaffected parents were obtained from the Simons Simplex Collection (SSC) as the controls (1). The access to the data was acquired from SSC on the National Institute of Mental Health Data Repository. Written informed consent was obtained from all participants as provided by the Simons Foundation Autism Research Initiative.

Exome sequencing

For CHD cases, DNA samples from venous blood or saliva were extracted and sequenced at the Yale Center for Genome Analysis as described before (2). Exomes were captured using Nimblegen v2 exome capture reagent, Nimblegen SeqxCap EZ MedExome Target Enrichment kit, or IDT xGen target capture kit, followed by Illumina DNA sequencing. 1,798 sibling-parent trios with WES from Simons Simplex Collection served as controls and were captured using Roche Nimblegen v2.

Sequence reads were aligned to the human reference genome GRCh37/hg19 by BWA-MEN. Single-nucleotide changes and small indels were called using a combined GATK Best Practice pipeline (3, 4) together with Freebayes (5) as described before (6). Variants were then annotated using ANNOVAR for allele frequencies in population databases gnomAD v2.1.1 (7) and Bravo (8), and deleteriousness by MetaSVM (9) and CADD v1.3 (10).

LoF variants were defined as stop-gain, stop-loss, frameshift insertion/deletion canonical splice site and start-loss variants. Damaging missense (D-Mis) were defined as missense variants predicted as “D”eleterious by MetaSVM or with a CADD v1.3 score of at least 30.

Variant filtering

Only high-quality variants that passed the GATK variant quality score calibration, with ≥ 8 read depth (DP), a genotype quality score (GQ) ≥ 20, and at least three supporting reads were identified. All recessive variants were visually inspected to remove false positives. Variants called in probands that had low-quality reads in parents were removed. Variants were filtered for minor allele frequency [MAF] ≤ 1×10^{-3} in gnomAD (v2.1.1) exome/genome and Bravo and only variants with ≤ 2 homozygotes in gnomAD were kept. LoF and D-Mis homozygous and compound heterozygous variants were considered for the recessive analysis. Homozygous variants were called from all probands. Compound heterozygous variants were called from trio samples. Variant pairs falling in the same genes in singleton probands were inferred to be compound heterozygotes if they were predicted to be in trans based on the absence of their co-occurrence in individuals in the gnomAD database using the tool (https://gnomad.broadinstitute.org/variant-cooccurrence). As a control, we called compound heterozygotes using this method in CHD probands from trios in our cohort in whom phase could be determined from parental genotypes. We found that calls of compound heterozygotes from these probands had 85% accuracy, consistent with expectation reported on the gnomAD website. RGs in *PLD1* and *C1orf127* were validated via Sanger sequencing.

Consanguinity determination

Beagle v3.3.2 (11) was used to perform haplotype phasing and the calculation of the inbreeding coefficient and the longest homozygosity-by-descent (HBD) fragment. People that are children from 1st, 2nd, 3rd, and 4th cousin unions should have an inbreeding coefficient centering around 0.0625, 0.0156, 0.0039, and 0.00098, respectively. By taking the midpoints of the two adjacent centers, we defined people from closer than 1st cousin unions as having an inbreeding coefficient ≥ 0.0313, people from 2nd cousin unions as 0.0078 to 0.0312, and people from 3rd cousin union as 0.0019 to 0.0077. By comparing the distribution of inbreeding coefficient in cases and controls (Figure S1), a more conserved cutoff 0.0009 to 0.0018 was used to define people from 4th cousin unions. We define consanguinity as people from closer than 4th cousin unions, that is having an inbreeding coefficient ≥ 0.0009, that is having homozygous segments of at least 2-centimorgan (cM) length comprising in total at least 0.09% of the genome.

Kinship analysis

For proband-parents trios, the relationship of three individuals in each family was examined using pairwise identify-by-descent (IBD) in PLINK (12). Trios with IBD sharing of 45-55% between probands and parents were considered as real trios.

We also examined the relatedness across all probands. Pairs of probands with PLINK PI_HAT score ≥ 0.2 or with ≥ 20% shared novel variants (MAF = 0% in gnomAD exome/genome, Bravo) were considered as related. Samples with the latest exome capture and the higher 8× sequence coverage were kept from each related pair.

Ancestry determination

The ancestry of each subject was determined using EIGENSTRAT software (13) as described (14). HapMap subjects were used as reference. The AJ ancestry was determined using LASER software (15). The genome-wide SNP array of 471 AJ individuals from the Gene Expression Omnibus database (16) and the 938 unrelated individuals from the Human Genome Diversity Project included in LASER were used as the reference. The reference samples were clustered in R and the ethnicity of the study samples were determined by comparing their coordinates to the reference.

Curation of known human recessive CHD gene list

The known human recessive CHD gene list was compiled based on the OMIM Human Genes and Genetic Disorders database and literature searches. 107/108 of the genes had associated CHD reported in OMIM. 104/108 were listed as having recessive inheritance, 4/108 did not have inheritance indicated in OMIM, but had literature reports of recessive inheritance and CHD. We included genes that either (1) showed genome-wide significance from an unbiased cohort study, OR (2) variants in the gene segregated with disease in at least 2 affected members of two or more families, OR (3) are genes underlying a recessive CHD-associated syndrome/disease with ≥ 2 cases with CHD, OR (4) is the only gene underlying a syndrome that has a strong clinical association with CHD, though there is no documentation of specific variants in two or more patients with the syndrome and CHD.

Gene enrichment analysis

The expected number of recessive variants was estimated as described previously (2). Specifically, we assumed that the expected frequency of damaging recessive variants in each gene would be proportional to the *de novo* mutability in each gene. The *de novo* mutability table was generated based on sequence context in correction of sequencing depth in each gene as described before (17). The table includes the mutation probabilities for 19,347 genes.

For an outbred population, the expected number of damaging RGs would be the square of the cumulative frequency of damaging alleles in each gene (q^2). For the inbred population, however, the expectation of compound heterozygotes in each gene increases as q^2, while the number of homozygotes increases linearly with q. We thus performed a polynomial regression analysis to get the relationship between the number of RGs and the *de novo* mutability (18) in each gene:

Number of RGs = $\beta_0 + \beta_1 \times mutabilty + \beta_2 \times mutabilty^2$

The expected number of RGs in each gene was then calculated using the fitted values from the polynomial model:

Expected number of $RG_i = N \times \frac{Fitted\ value_i}{\sum_{Genes} Fitted\ value}$, where i denotes the ith gene and N denotes the total number of RGs.

Similarly, for a given gene set, the expected number of RGs was estimated as:

Expected number of $RG_{Gene\ set} = N \times \frac{\sum_{Gene\ set} Fitted\ value}{\sum_{Genes} Fitted\ value}$

Then, a one-tailed binomial test was used to compare the significant difference between the number of observed and the expected RGs in each gene or a given gene set. The Bonferroni multiple testing threshold is 2.6×10^{-6} (0.05/19,347) for the gene-based test.

The number of genes with more than one RG was estimated using a permutation test as described before (2). The total number of observed RGs in each functional category was randomly distributed onto the genome using the fitted values from the polynomial model. One million permutations were performed. The P-value was calculated as the proportion of times that we observed ≥ 2 RGs in a given number of genes among all permutations.

Single-cell RNA-seq analysis

The single-cell RNA-seq data of mouse gastrulation was acquired from Pijuan-Sala et al (19). The mouse gene names were matched to their human homologue through bioMart (20) and manual inspections. 17,273 out of the 19,347 human genes from the mutability table were matched and have non-zero expression in the dataset. For human genes with multiple mouse homologues, the average expression was used. The expression of a gene in a certain tissue was normalized through dividing it by the highest expression of that gene across all tissue types (ratio of the maximum expression).

One hundred fifty-two genes harbored at least two RGs in the cohort. The 141 out of 152 genes with available expression data were clustered by their ratio of max expression in different tissue types using UPGMA hierarchical clustering algorithm through python package seaborn v0.11.0.

A gene is considered as specifically expressed in one tissue if its expression is at least five-fold higher in that tissue compared to any other tissues.

Recessive contribution estimation

The contribution of the recessive genotypes to the CHD in the cohort was estimated, as previously described (17). Specifically, the number of expected RGs in probands was estimated from the frequency of rare (MAF < 0.001) likely damaging variants in parents in each gene, corrected for runs of homozygosity (ROH). To estimate the ROH, only high-quality SNPs with < 2% missing values and with a minimum allele frequency of 1% across the entire cohort of 3,787 trios were considered. The data were then pruned to exclude SNPs in strong linkage disequilibrium in order to avoid overcalling of ROH. The autozygosity regions were called using bcftools/roh (v1.9) (21). The r^2 cutoff and the quality score cutoff for estimating segments of autozygosity were picked to make the ratio of the observed to expected synonymous RGs close to 1.

For calculation of the in-cohort allele frequency and to avoid overestimation of expected RGs from false-positive variants, only the variants that passed the following criteria were kept: GQ ≥ 20, DP ≥ 8, Mapping Quality score [MQ] ≥ 40, variant allele fraction ≥ 25%, at least 3 supporting reads, and not in SegDup regions in all three members of a trio.

When estimating RG expectation in phenotype subsets, in-cohort allele frequency, LD pruning, and ROH calculation were all based on samples within each sub-phenotype.

Afterward, the proportion of probands (π) with causal variants of a specific variant class was estimated using both probands and parental data as described (17).

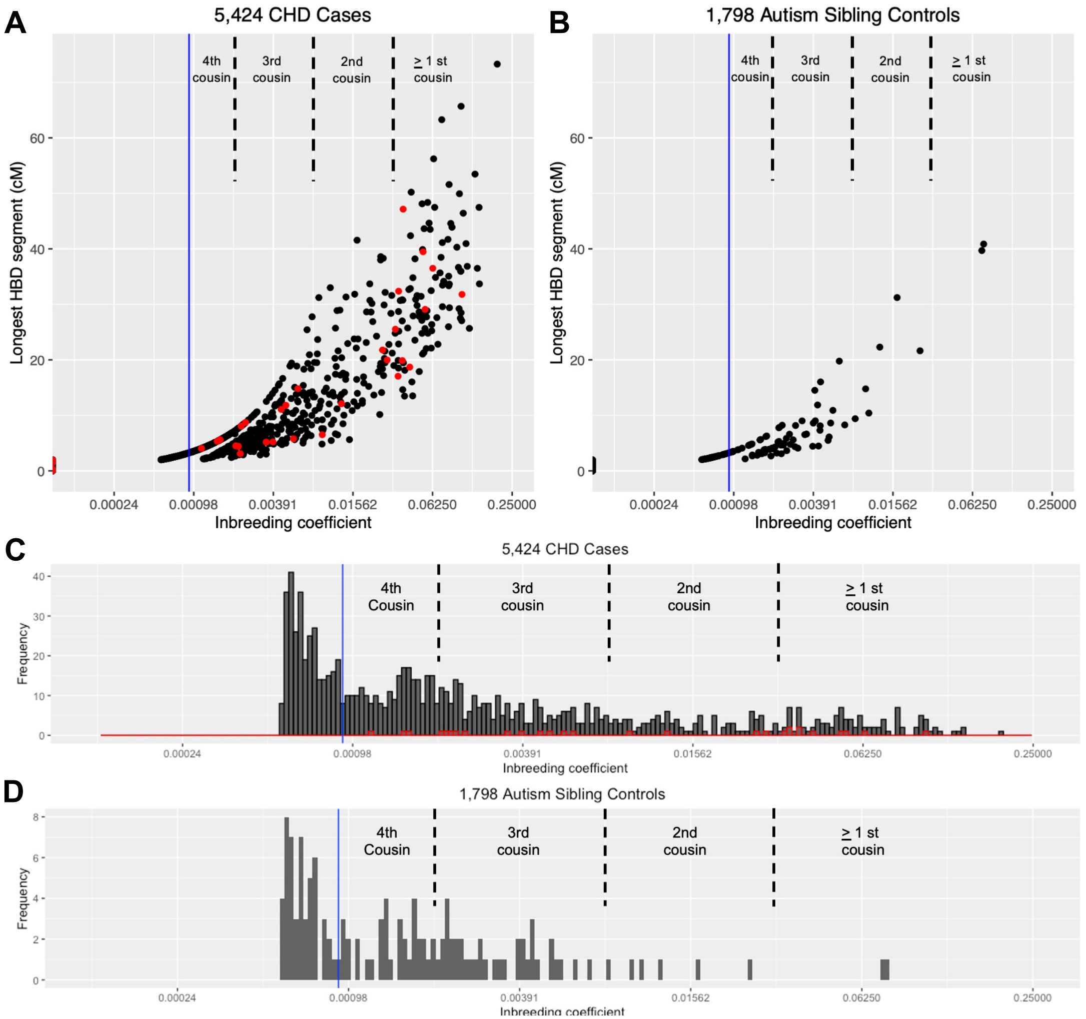

Fig. S1. Determination of consanguinity by inbreeding coefficient.

Longest HBD segment vs inbreeding coefficient plot for (A) 5,424 CHD cases and (B) 1,798 autism sibling controls. Distribution of inbreeding coefficient for (C) 5,424 CHD cases and (D) 1,798 autism sibling controls. The x-axises are of log4 scale. People that are children from 1st, 2nd, 3rd, and 4th cousin unions should have an inbreeding coefficient centering around 0.0625, 0.0156, 0.0039, and 0.00098, respectively. By taking the midpoints (black dash lines) of the two adjacent centers, we defined people from closer than 1st cousin unions as having an inbreeding coefficient ≥ 0.0313, people from 2nd cousin unions as 0.0078 to 0.0312, and people from 3rd cousin union as 0.0019 to 0.0077. By comparing the distribution of inbreeding coefficient in cases (C) and controls (D), a more conserved cutoff 0.0009 to 0.0018 was used to define people from 4th cousin unions. We define consanguinity as people from closer than 4th cousin unions, that is having an inbreeding coefficient ≥ 0.0009 (blue lines). Red dots indicate CHD cases with homozygous variants in known human recessive CHD genes. An outlier of CHD case with longest HBD segment of 109.5 and inbreeding coefficient of 0.049 was not shown in the graph. In (C) and (D), 4449 cases and 1669 controls with inbreeding coefficient of 0 are not shown.

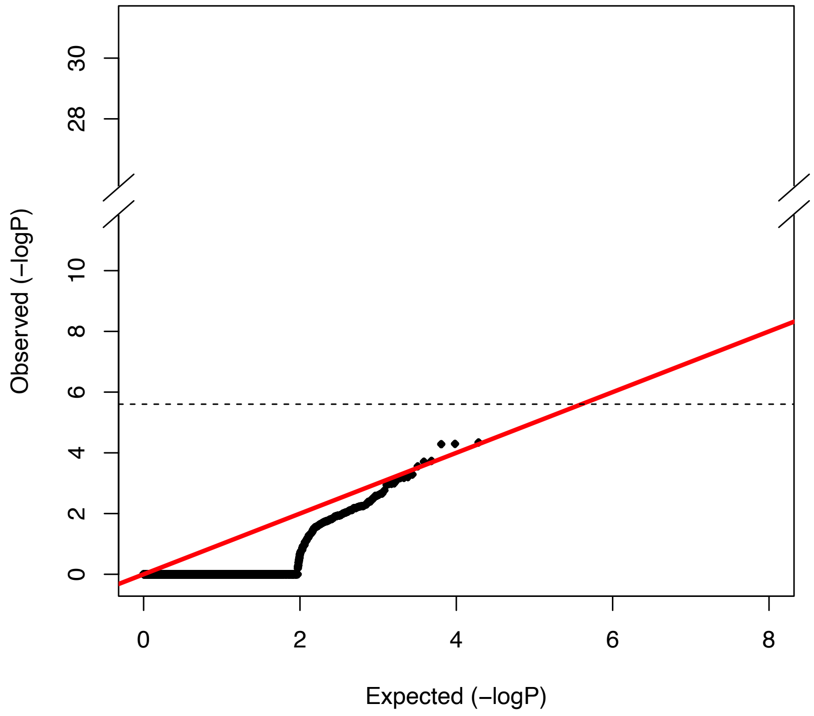

Fig. S2. Binomial test of damaging recessive genotypes in 1,798 controls.

No significant enriched genes were found for LoF RGs in controls. Dashed lines indicate the genome-wide significant p-value cutoff: 2.6×10^{-6} (0.05/19347).

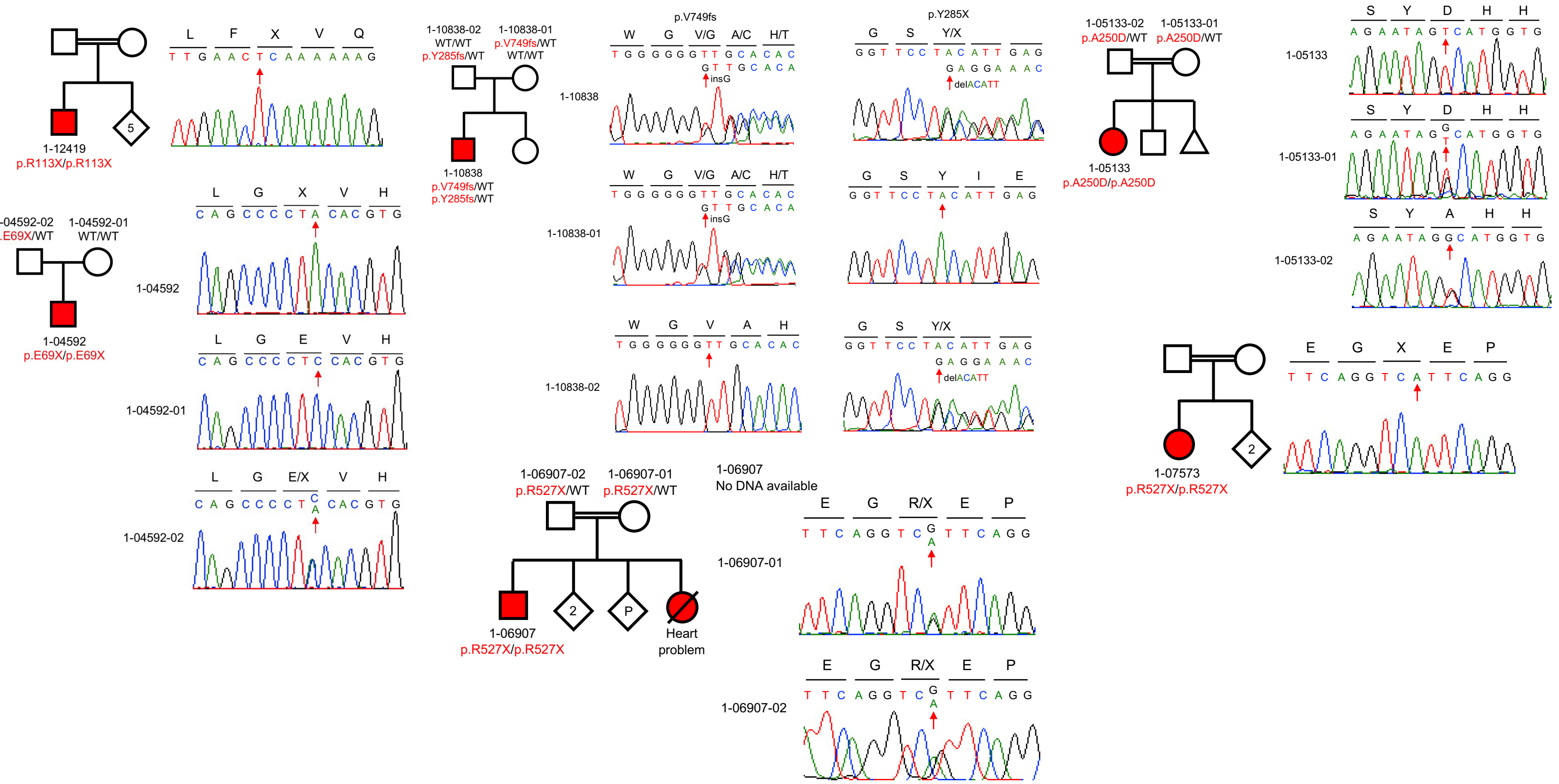

Fig. S3. Pedigrees and variants in *C1orf127* kindreds.

Mutated bases and the corresponding wild-type bases are marked with arrows on the chromatograms. Red: with heart disease; Square: males; Circle: females; Slash: deceased; Triangle: fetal death; Diamond: siblings; Number at the center of the diamond: number of siblings. Triangle with P at the center: in gestation.

A

Gene	# Observed RGs	# Expected RGs	Enrichment	P-Val	pRec	Heart Expr %Rank
C1orf127	**5**	**1.27E-02**	**392.4**	**2.62E-12**	**0.56**	**35.2**
USP17L7	2	6.28E-03	318.6	1.95E-05	NA	NA
CD36	2	6.73E-03	297.0	2.24E-05	0.00	84.7
ZNF844	2	7.39E-03	270.5	2.70E-05	0.52	NA
PPEF2	2	1.16E-02	172.0	6.67E-05	0.65	16.3
KEL	2	1.36E-02	147.6	9.05E-05	0.03	35.2
MYO1A	2	1.79E-02	111.7	1.58E-04	0.00	30.4
ANKRD36	2	2.64E-02	75.8	3.40E-04	0.00	NA
PKD1L1	2	3.22E-02	62.1	5.05E-04	1.00	15.5
SYNM	1	2.93E-03	340.9	2.93E-03	0.00	58.9

B

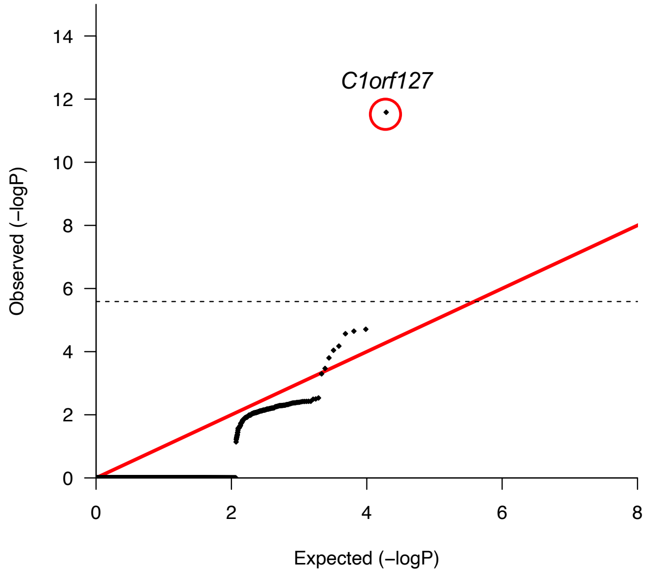

C

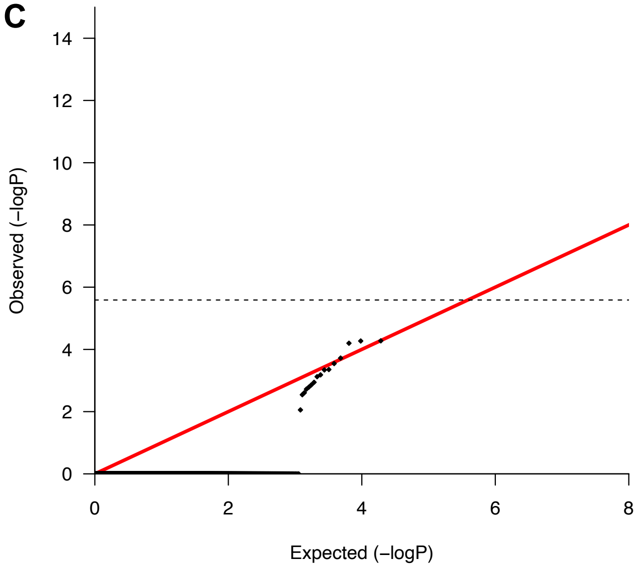

Fig. S4. Binomial test of LoF recessive genotypes in 5,424 CHD cases.

(A) Top 15 genes from the binomial test in 5,424 CHD cases. The gene with genome-wide significant enrichment was bolded ($P < 2.6 \times 10^{-6}$ [0.05/19347]). (B) Q-Q plot comparing observed versus expected *P* values for damaging RGs in cases. (C) No significant enriched genes were found for LoF RGs in 1,798 controls.

A

Gene	# Observed RGs	# Expected RGs	Fold Change	P-Val	pRec	Heart Expr %Rank
C1orf127	**4**	**4.18E-03**	**957.8**	**1.12E-11**	**0.56**	**35.2**
PKD1L1	2	1.23E-02	162.7	7.35E-05	1.00	15.5
SYNM	1	1.08E-04	9270.3	1.08E-04	0.00	58.9
PATE1	1	4.42E-04	2263.9	4.42E-04	0.02	0.0
OR8H3	1	4.63E-04	2159.8	4.63E-04	0.65	0.0
OR52N5	1	4.99E-04	2003.2	4.99E-04	0.27	0.0
OR2T27	1	6.93E-04	1443.6	6.92E-04	0.12	NA
RLN1	1	8.59E-04	1163.7	8.59E-04	0.53	15.9
TMEM91	1	8.77E-04	1139.8	8.77E-04	0.80	25.5
FAM151B	1	9.96E-04	1004.0	9.96E-04	0.20	31.7

B

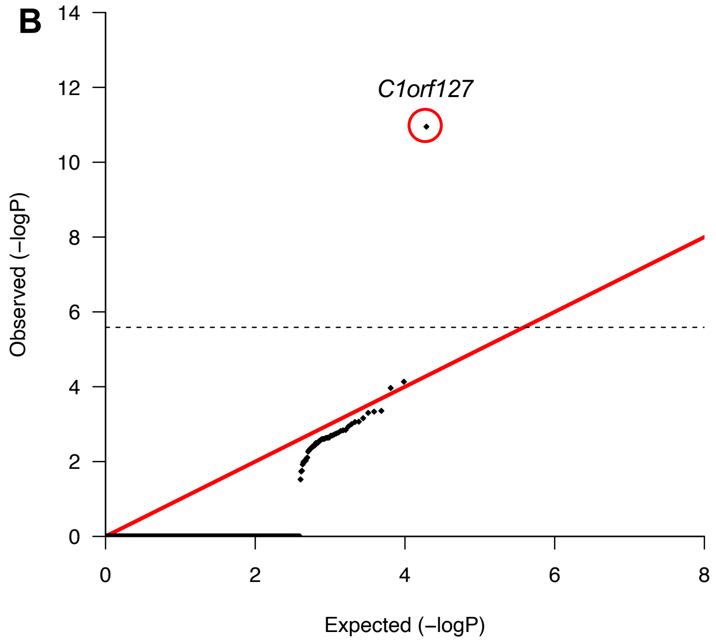

Fig. S5. Binomial test of LoF recessive genotypes in 1,160 cases with laterality-associated defect.
(A) Top 15 genes from the binomial test. The gene with genome-wide significant enrichment was bolded ($P < 2.6 \times 10^{-6}$ [0.05/19347]). (B) Q-Q plot comparing observed versus expected *P* values for damaging RGs.

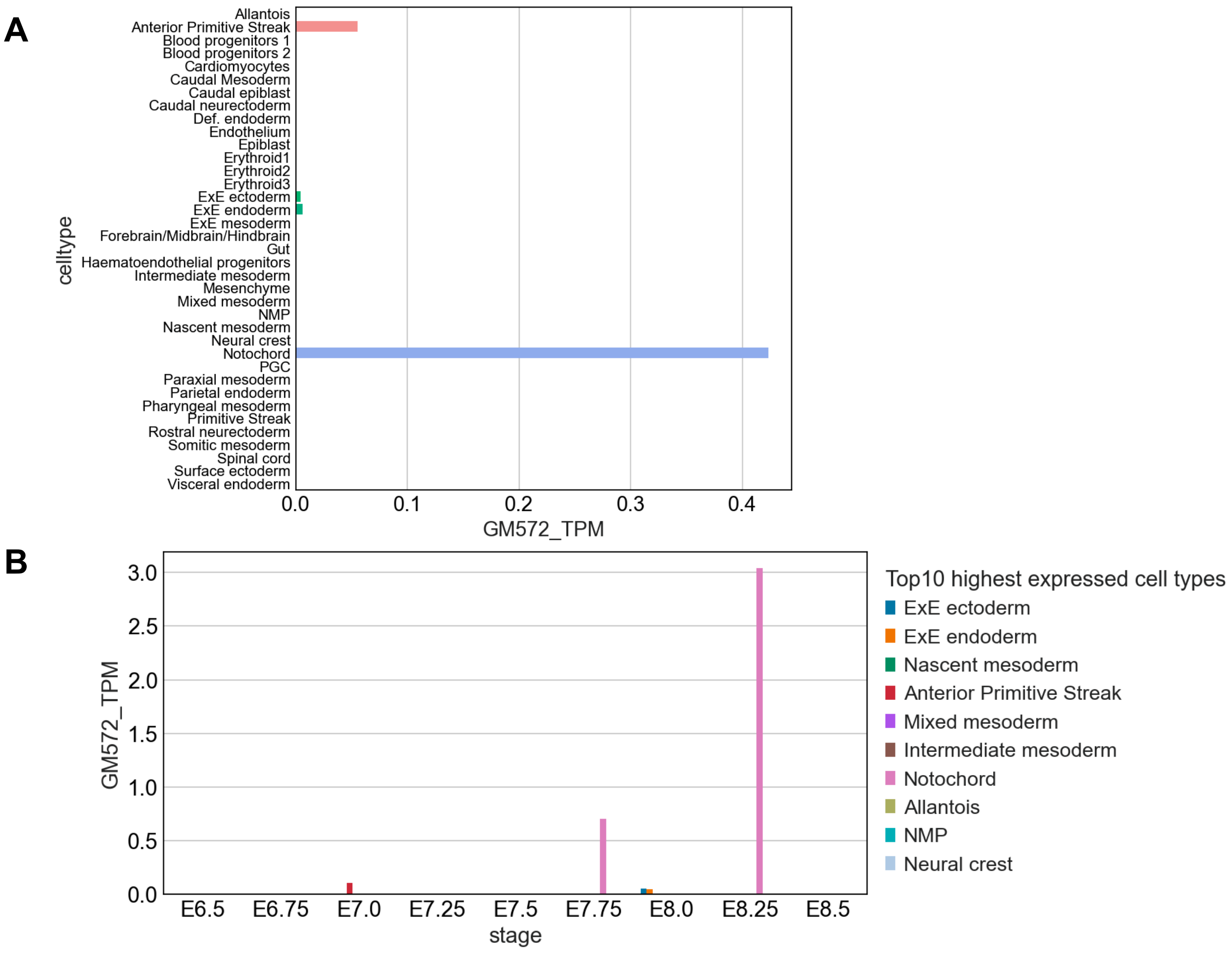

Fig. S6. *Gm572* expression during mouse gastrulation.

Gm572 expression in (A) different cell types and in (B) different developmental stages based on Pijuan-Sala et al.'s mouse gastrulation single-cell RNA-seq dataset (doi: 10.1038/s41586-019-0933-9).

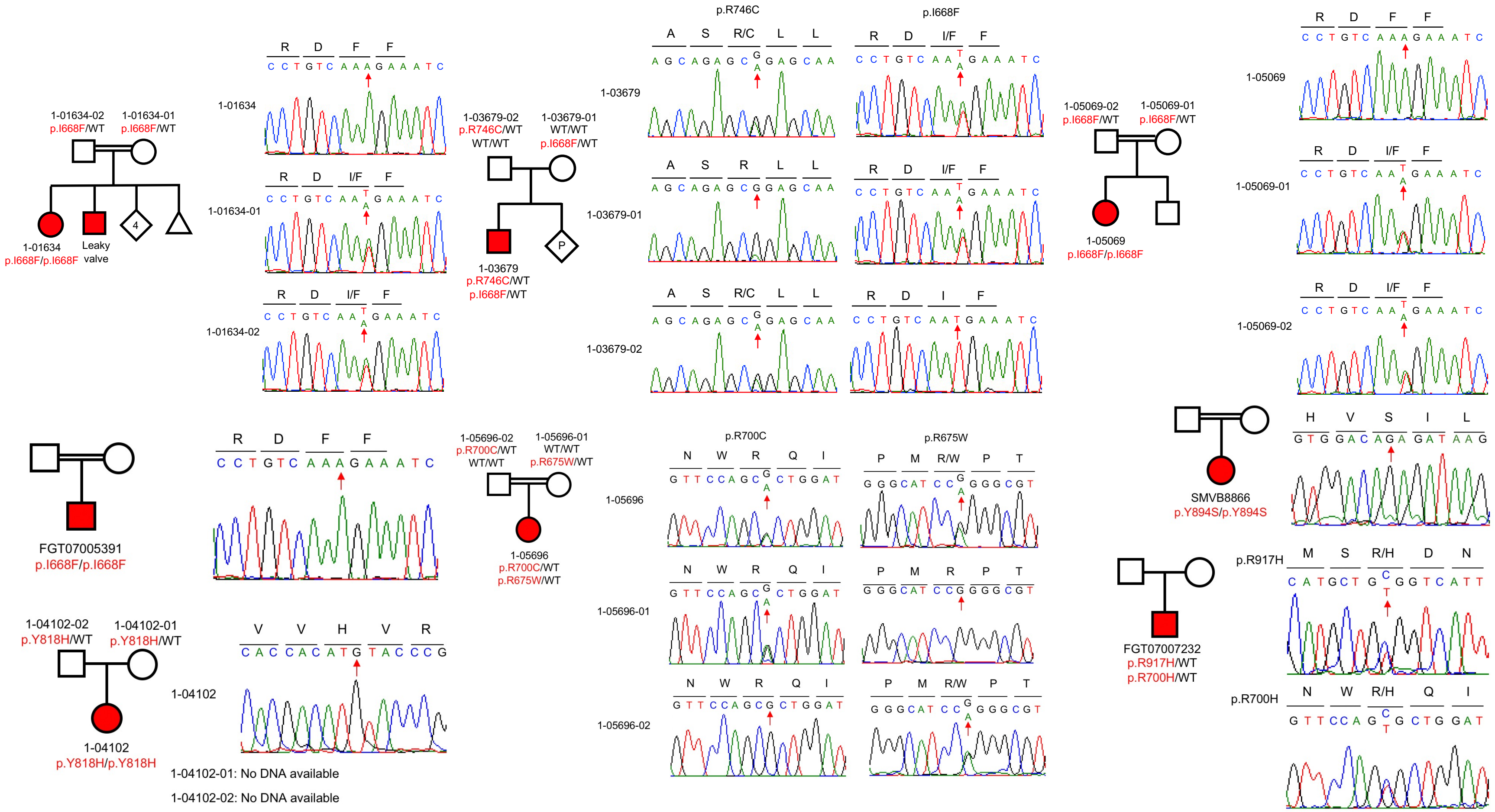

Fig. S7. Pedigrees and variants in *PLD1* kindreds.

Mutated bases and the corresponding wild-type bases are marked with arrows on the chromatograms. Red: with heart disease; Square: males; Circle: females; Slash: deceased; Triangle: fetal death; Diamond: siblings; Number at the center of the diamond: number of siblings. Triangle with P at the center: in gestation.

A

Gene	# Observed RGs	# Expected RGs	Fold Change	P-Val	pRec	Heart Expr %Rank
PLD1	**6**	**8.73E-03**	**686.9**	**5.10E-16**	**0.86**	**51.4**
FFAR1	1	1.12E-04	8933.0	1.12E-04	0.55	0.0
H6PD	2	1.64E-02	122.2	1.31E-04	0.91	70.4
HKDC1	2	2.38E-02	84.0	2.76E-04	0.92	18.0
CFTR	2	2.48E-02	80.6	3.00E-04	0.00	24.0
SHISA3	1	3.95E-04	2530.1	3.95E-04	0.39	21.7
C7orf25	1	4.57E-04	2187.3	4.57E-04	0.64	44.7
PXMP2	1	1.25E-03	797.3	1.25E-03	0.82	42.6
SSBP4	1	1.59E-03	630.6	1.58E-03	0.63	68.4
EFNA4	1	2.46E-03	407.3	2.45E-03	0.62	44.7

B

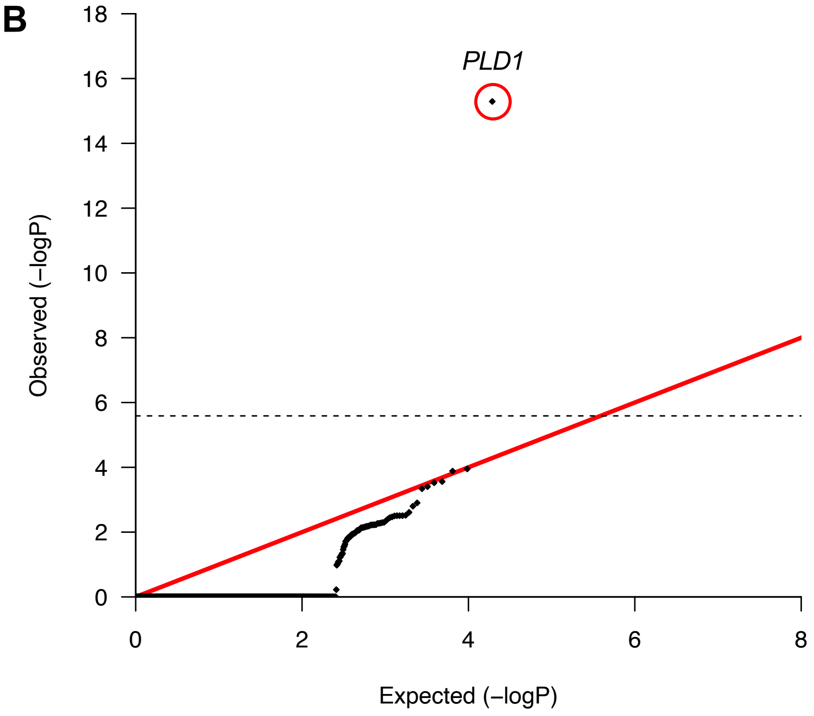

Fig. S8. Binomial test of damaging recessive genotypes in 330 cases with right-sided valvular defects.
(A) Top 10 genes from the binomial test. The gene with genome-wide significant enrichment was bolded ($P < 2.6 \times 10^{-6}$ [0.05/19347]). (B) Q-Q plot comparing observed versus expected *P* values for damaging RGs.

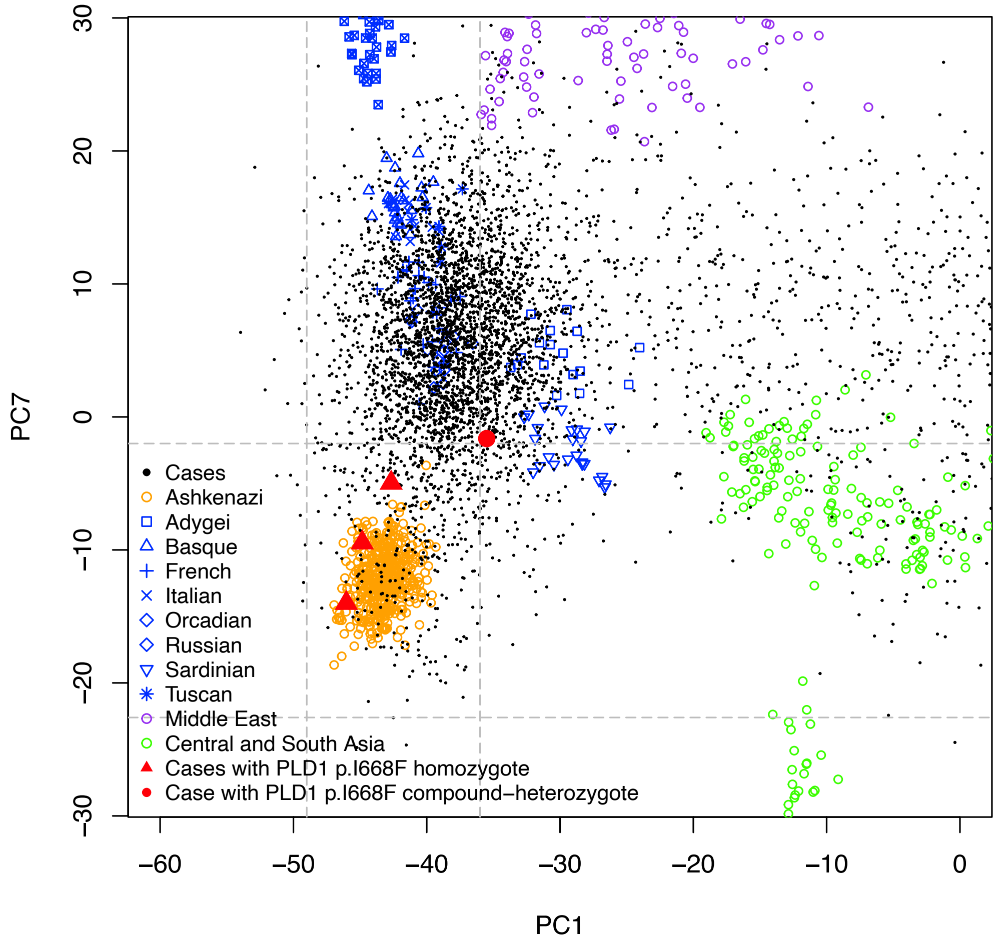

Fig. S9. Principle component analysis for ancestry of probands with *PLD1* p.I668F variant.

410 (7.6%) CHD probands cluster with Ashkenazim (black dots). The three patients with *PLD1* p.Ile668Phe homozygotes were denoted by red triangles while the one patient with *PLD1* p.Ile668Phe compound-heterozygote was denoted by the red circle (from top to bottom: 1-03679, 1-01634, 1-05069, FGT07005391).

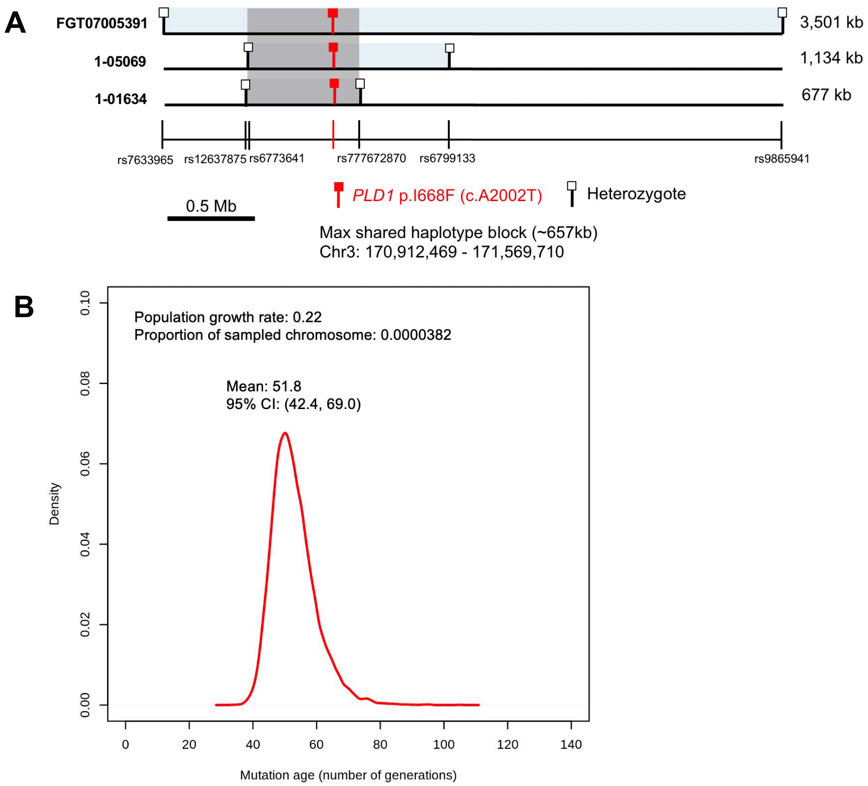

Fig. S10. Haplotype sharing and age estimation for three *PLD1* p.Ile668Phe (c.A2002T) homozygotes.

(A) A 3.5-Mb segment of chromosome 3 across the location of the homozygous *PLD1* p.Ile668Phe (c.A2002T; red square) in three mutated subjects were shown. The closest heterozygous SNP to each side of *PLD1* p.Ile668Phe (c.A2002T) in each subject is shown as a white square. Tick marks at the bottom indicate locations of all SNPs found on the segment. The max shared haplotype block across the three samples is 657kb (shown as gray vertical bar). (B) Variant age (the number of generations, x-axis) was estimated using DMLE+2.3 with a total of 1,000,000 iterations. The y-axis indicates the frequency of occurrences of each variant age. The population growth rate (PGR) and proportion of sampled chromosome (PSC) for *PLD1* p.Ile668Phe in Ashkenazi Jewish were estimated as 0.22 and 0.0000382, respectively. The average variant age is 51.8 and the 95% confidence interval is between 42.4 and 69.0.

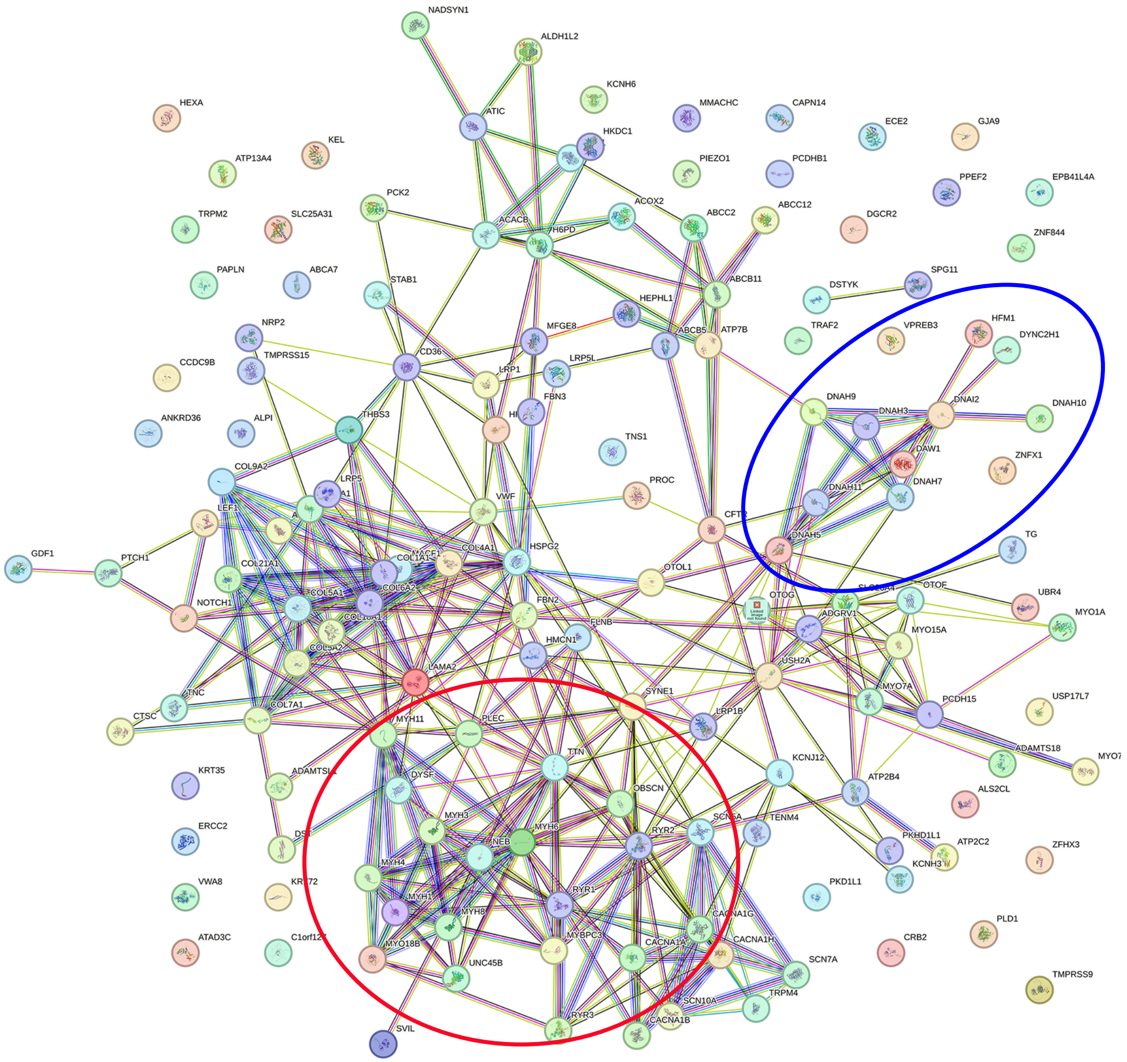

Fig. S11. STRING analysis of cilia and sarcomere proteins.

The cluster containing cilia proteins is outlined in blue and the cluster containing sarcomere proteins is outlined in red.

Table S1. CHD Cohort Characteristics.

	Cases	Controls
**Ancestry**		
European	3875 (71.4%)	1418 (78.9%)
African American	339 (6.3%)	77 (4.3%)
Mexican	523 (9.6%)	129 (7.2%)
South Asian	209 (3.9%)	88 (4.9%)
East Asian	116 (2.1%)	40 (2.2%)
Undetermined	362 (6.7%)	46 (2.6%)
**Gender**		
Males	3157 (58.2%)	843 (46.9%)
Females	2267 (41.8%)	955 (53.1%)
**CHD Phenotype**		
Heterotaxy (HTX)	535 (9.9%)	NA
Conotruncal defect (CTD)	1837 (33.9%)	NA
Tetralogy of Fallot (TOF)	932 (17.2%)	NA
Left ventricular obstruction (LVO)	1383 (25.5%)	NA
Hypoplastic left heart syndrome (HLHS)	844 (15.6%)	NA
D-transposition of the great arteries (D-TGA)	385 (7.1%)	NA
Other	1154 (21.3%)	NA
Atrial septal defect (ASD)	222 (4.1%)	NA
Atrioventricular canal (AVC)	208 (3.8%)	NA
Unknown	130 (2.4%)	NA
**Other Phenotype Grouping**		
Laterality-associated defect	1160 (21.4%)	NA
Left-sided defect	1849 (34.1%)	NA
Right-sided valvular defect	330 (6.1%)	NA
**Proband Family Structures**		
Parent-Affected Offspring Trios	3716 (68.5%)	1798 (100.0%)
Singleton probands	1708 (31.5%)	0 (0.0%)
**Total**	**5424**	**1798**

Heterotaxy (HTX) includes situs abnormalities such as dextracardia, left or right isomerism as the major malformation and may include other defects such as L-transposition of the great arteries (L-TGA), atrioventricular canal defects (AVC), anomalous pulmonary venous drainage (TAPVR, PAPVR), and double outlet right ventricle (DORV). Conotruncal defect (CTD) includes tetralogy of Fallot (TOF), DORV, truncus arteriosus, membranous ventricular septal defects (VSD), and aortic arch abnormalities. Left ventricular obstruction (LVO) includes hypoplastic left heart syndrome (HLHS), coarctation of the aorta (CoA), and aortic stenosis/bicuspid aortic valve (AS/BAV). Other phenotypes include pulmonary valve abnormalities, anomalous pulmonary venous drainage, atrial septal defects (ASD), atrioventricular canal defects, double inlet left ventricle (DILV), and tricuspid valve atresia (TA). Laterality-associated defect is HTX, D-TGA or DORV. Left-sided defect includes mitral valve defect, aortic coarctation, aortic stenosis, or aortic valve defects. Right-sided valvular defect includes tricuspid atresia, Ebstein, pulmonary atresia without ASD, or hypoplastic right ventricle with tricuspid valve defect.

Table S2. Sequencing metrics.

Category	Cases (Roche V2; N=5,435)	Cases (MedExome; N=3,019)	Cases (xGen IDT; N=4,402)	Controls (Roche V2; N=5,394)
Read length (bp)	73-76	76-101	101	50-99
# of reads per sample (M)	76.0	53.9	55.4	111.5
Median coverage at each targeted base (X)	63.9	35.7	58.6	68.0
Mean coverage at each targeted base (X)	77.1	41.5	61.8	80.8
% of all reads that map to target	63.5%	47.5%	57.2%	46.5%
% of all bases that map to target	47.7%	37.7%	42.8%	35.7%
% of targeted bases read at least 8x	93.4%	96.4%	98.5%	92.7%
% of targeted bases read at least 10x	92.0%	95.1%	98.3%	91.4%
% of targeted bases read at least 15x	88.1%	89.6%	97.3%	87.8%
% Mean error rate	0.3%	0.4%	0.3%	0.5%

Table S3. Frequency of homozygous RGs in CHD probands increases with inbreeding coefficient.

	**Inbreeding coefficient range**	**Cases (N=5242)**			**Controls (N=1798)**
		# (%) Individuals	# Homozygotes in known human recessive CHD genes	% Individuals with homozygotes in known human recessive CHD genes	# (%) Individuals
≥ 1st cousin	Inbreeding coefficient ≥ 0.0313	105 (1.9%)	10	9.5%	2 (0.1%)
2nd cousin	Inbreeding coefficient 0.0078 - 0.0312	102 (1.9%)	4	3.9%	6 (0.3%)
3rd cousin	Inbreeding coefficient 0.0019 - 0.0077	244 (4.5%)	11	4.5%	39 (2.2%)
4th cousin	Inbreeding coefficient 0.0009 - 0.0018	224 (4.1%)	3	1.3%	33 (1.8%)
More distant than 4th cousin	Inbreeding coefficient < 0.0009	4749 (87.6%)	4	0.08%	1718 (95.6%)

Table S4. Genotype-phenotype concordance of recessive genotypes in known CHD genes.

(A) Overall genotype-phenotype concordance

**# Total RGs**	66
**# Concordant**	43 (65.2%)
**# Intermediate**	17 (25.8%)
**# Not concordant**	4 (6.1%)
**# Unknown**	2 (3.0%)

(B) Genotype-phenotype concordance in individual genes

Gene	Cilia genes	# Total RGs	# Concordant	# Intermediate	# Not concordant	# Unknown
GDF1		13	11	2	0	0
MYH6		12	9	0	2	1
DNAH5	Yes	7	3	4	0	0
PLD1		6	6	0	0	0
DNAH9	Yes	3	1	2	0	0
DNAI2	Yes	3	1	2	0	0
ATIC		2	1	0	1	0
DNAH11	Yes	2	1	1	0	0
DYNC2H1	Yes	2	1	1	0	0
NADSYN1		2	2	0	0	0
PKD1L1	Yes	2	2	0	0	0
ARMC4	Yes	1	1	0	0	0
CCDC39	Yes	1	0	1	0	0
COL1A2		1	0	0	1	0
CTU2		1	0	0	0	1
DNAAF1	Yes	1	1	0	0	0
EVC	Yes	1	0	1	0	0
KYNU		1	1	0	0	0
LZTR1		1	0	1	0	0
MMP21		1	1	0	0	0
NPHP3	Yes	1	0	1	0	0
NPHP4	Yes	1	1	0	0	0
PIGN		1	0	1	0	0

Table S5. Enrichment of damaging recessive genotypes in different CHD subtypes

Diagnosis	# Probands	# RGs	# Observed RGs in CHD genes (%)	# Expected RGs in CHD genes	Enrichment	P-Val
**HTX**	535	152	18 (3.4%)	1.8	9.9	**$5.5{\times}10^{-13}$**
**D-TGA**	385	62	5 (1.3%)	0.8	6.0	**$1.6{\times}10^{-3}$**
**LVO**	1383	266	13 (0.9%)	3.7	3.5	**$1.1{\times}10^{-4}$**
HLHS	844	142	8 (1.0%)	2.0	4.0	**$1.1{\times}10^{-3}$**
**CTD**	1837	510	20 (1.1%)	7.0	2.9	**$3.8{\times}10^{-5}$**
TOF	932	242	8 (0.9%)	3.4	2.4	$2.2{\times}10^{-2}$
**Other**	1154	249	10 (0.9%)	3.0	3.3	**$1.1{\times}10^{-3}$**
ASD	222	56	1 (0.5%)	0.5	1.8	$4.2{\times}10^{-1}$
AVC	208	44	4 (1.9%)	0.6	6.5	**$3.3{\times}10^{-3}$**
**Laterality-associated defect**	1160	258	28 (2.4%)	3.8	7.4	**$5.5{\times}10^{-16}$**

Enrichment of damaging RGs in known human recessive CHD genes in different CHD subtypes. Enrichment and p-values for known human recessive CHD genes were calculated using the binomial test adjusted for fitted values from the polynomial model using damaging *de novo* probabilities. The cutoff for significant p-values is 0.0056 = 0.05/9 after multiple testing correction. The significant p-values are bolded.

Table S6. Enrichment of recessive genotypes in cilia genes.

Samples	Observed				Expected	Enrichment	P-value
	# Hom	# CompHet	# Unique genes	# RGs	# RGs		
**358 Cilia Genes**							
All 5424 Cases	22	57	45	79	51.14	1.54	1.30E-04
1160 Laterality-Associated Defect Cases	7	17	19	24	11.63	2.06	7.54E-04
4134 Non-Laterality-Associated Defect Cases	15	37	33	52	38.57	1.35	2.02E-02
**42 Cilia Genes that are also Known Human Recessive CHD genes**							
All 5424 Cases	8	17	12	25	9.82	2.54	3.25E-05
1160 Laterality-Associated Defect Cases	3	10	9	13	2.07	6.29	2.47E-07
4134 Non-Laterality-Associated Defect Cases	5	7	7	12	7.54	1.59	8.08E-02

Cilia genes are based on Syscilia gene set (doi: 10.1186/2046-2530-2-7).

Table S7. Additional RGs in *GDF1* and *MYH6*.

ID	Sex	Ancestry	Consanguin-eous?	Phenotype	Diagnosis	Gene	Mutation Type	cDNA Change	Amino Acid Change	gnomAD WES Freq	gnomAD WGS Freq	Bravo Freq	Meta SVM	CADD
1-06272	F	European	No	HTX	Heterotaxy, Situs ambiguous, Asplenia, Anomalous hepatic venous drainage or connection, ASD, DORV, AVC, Hypoplastic LV, Hypoplastic main pulmonary artery, IVC anomaly, Left aortic arch with normal branching pattern, LSVC entering LA, LSVC - communicating with RSVC, PDA, PA, PS, Pulmonary vein anomaly, RSVC to RA	*GDF1*	CompHet	c.909dupC	p.V304fs	2.00E-04	9.00E-04	3.19E-05	N/A	N/A
								c.T392C	p.F131S	0	0	0	D	27.1
1-08845	M	European	Yes	HTX	Dextracardia, L-TGA, VSD, PS, MAPCAS, PA	*GDF1*	Hom	c.T1091C	p.M364T	5.00E-04	1.00E-04	1.99E-04	D	26.9
1-01768	M	African	Yes	LVO (HLHS)	Aortic atresia, HLHS, Hypoplastic LV, LSVC to coronary sinus, Mitral atresia, Tubular hypoplasia of aorta, CoA	*MYH6*	Hom	c.G831T	p.Q277H	4.00E-04	1.00E-04	2.31E-04	D	21.9
1-14851	F	European	Yes	Other	CoA, Single LV, DILV,	*MYH6*	Hom	c.C3995T	p.A1332V	0	0	7.96E-06	D	27.8
S48AWOU4	F	European	No	Other	Simple shunt lesions (e.g., ASD, VSD, PAPVR, PDA), repaired/small without sequelae | ASD	*MYH6*	Hom	c.C1702T	p.R568C	3.66E-05	3.23E-05	5.57E-05	D	31
SDVCC21O	F	European	Yes	CTD	Simple shunt lesions with Eisenmenger syndrome | Eisenmenger physiology	*MYH6*	Hom	c.G3469A	p.G1157R	1.28E-05	0	7.96E-06	D	33
1-06040	M	European	No	OTH (AVC)	Balanced transitional atrioventricular canal | Cleft mitral valve | Left aortic arch with normal branching pattern	*MYH6*	Inferred CompHet	c.C4136T	p.T1379M	6.00E-04	4.00E-04	5.02E-04	D	26.5
								c.C3893T	p.A1298V	1.00E-04	4.00E-04	1.19E-04	D	27.8
FGT07005303	F	European	No	LVO (HLHS)	Hypoplastic left heart syndrome | Aortic stenosis and mitral stenosis | Intact ventricular septum	*MYH6*	Inferred CompHet	c.3859+1G>T	NA	4.06E-06	.	.	.	25.9
								c.G831T	p.Q277H	4.00E-04	1.00E-04	2.31E-04	D	21.9

ASD: atrial septal defect; DORV: double-outlet right ventricle; AVC: atrioventricular canal; LV: left ventricle; RV: right ventricle; IVC: inferior vena cava; LSVC: left superior vena cava; RSVC: right superior vena cava; LA: left atrium; PDA: patent ductus arteriosus; PA: pulmonary atresia; PS: pulmonary stenosis; L-TGA: L-transposition of great artery; VSD: ventricular septal defect; MAPCAS: multiple major aortopulmonary collateral arteries; HLHS: hypoplastic left heart syndrome; CoA: Coarctation of aorta; DILV: double-inlet left ventricle.

Table S8. Damaging RGs in NAD biosynthetic genes.

ID	Trio?	Sex	Age	Ancestry	Consanguin-eous?	Phenotype	Cardiac	Extracardiac	Gene	Mutation Type	cDNA Change	Amino Acid Change	gnomAD WES Freq	gnomAD WGS Freq	Bravo Freq	Meta SVM	CADD
1-06907	Yes	M	10y, 6m	South Asian	Yes	CTD	Dilated left atrium, Ventricular defect uncommitted DORV, Hypoplastic MV, PFO	Cleft palate, Tracheoesophageal fistula, Congenital abnormality of the supraglottis, Congenital laryngeal anomaly, Chronic lung disease, Inguinal hernia, Congenital craniocervical deformity of the spine with massive head tilt	*NADSYN1*	Hom	c.G1717A	p.A573T	7.00E-04	1.20E-03	8.28E-04	D	27.4
1-15292	Yes	F	N/A	European	No	LVO (HLHS)	Secundum ASD, Mitral stenosis	N/A	*NADSYN1*	CompHet	c.G1621A<br>c.G1717A	p.G541R<br>p.A573T	1.62E-05<br>7.00E-04	3.24E-05<br>1.20E-03	7.17E-05<br>8.28E-04	T<br>D	33<br>27.4
GT04010762	Yes	M	N/A	European	No	Other (AVC)	AVC	N/A	*NADSYN1*	Hom	c.G1717A	p.A573T	7.00E-04	1.20E-03	8.28E-04	D	27.4
1-03667	No	F	3y, 7m	South Asian	No	LVO (HLHS)	Aortic stenosis | Hypoplastic left heart syndrome | Left superior vena cava connecting to the coronary sinus | Mitral stenosis	N/A	*NADSYN1*	Inferred CompHet	c.85+1G>C<br>c.G395T	splice<br>p.W132L	1.65E-05<br>5.00E-04	.<br>9.69E-05	2.39E-05<br>1.83E-04	.<br>D	26.1<br>26.1
9504000282-0	No	M	N/A	Mexican	No	OTH (AVC)	Straddling MV with LV hypoplasia and outflow obstruction	N/A	*NADSYN1*	Inferred CompHet	c.G395T<br>c.C1036T	p.W132L<br>p.R346X	5.00E-04<br>2.44E-05	9.69E-05<br>.	1.83E-04<br>3.98E-05	D<br>.	26.1<br>41.0
1-00222	Yes	F	1m	Undetermined	Yes	LVO (HLHS)	Aortic atresia, Secundum ASD, CoA, HLHS, LSVC to coronary sinus, Mitral atresia	Gastroesophageal reflux, spina bifida, Congenital hemivertebra	*KYNU*	Hom	c.T1112C	p.L371P	4.07E-06	0	7.96E-06	D	25.9
1-04832	Yes	M	5y, 4m	European	Yes	LVO (HLHS)	Aortic atresia, HLHS (subnormal cavity volume), LSVC non-communicating with RSVC, Mitral atresia, Tubular hypoplasia of aorta	Bifid uvula, Single kidney, Undescended testis	*KMO*	Hom	c.T644A	p.I215N	0	0	0	D	32

CTD: conotruncal defect; DORV: double-outlet right ventricle; MV: mitral valve; PFO: patent foramen ovale; ASD: atrial septal defect; AVC: atrioventricular canal; LVO: left ventricular outflow tract obstruction; CoA: coarctation of aorta; HLHS: hypoplastic left heart syndrome; LSVC: persistent left superior vena cava; RSVC: right superior vena cava.

Table S9. *PLD1* recessive genotypes and phenotypes.

ID	Sex	Ancestry	Consanguin-eous?	Detailed Cardiac Phenotype	Mutation Type	cDNA Change	Amino Acid Change	Bravo Freq	Meta SVM	CADD
#1-05069	F	European (ASJ)	Yes	Hypoplastic RV, Pulmonary atresia, Tricuspid stenosis, VSD	Hom	c.A2002T	p.I668F	6.05E-04	T	34
#1-01634	F	European (ASJ)	Yes	Coronary anomaly, Hypoplastic RV, Pulmonary atresia, Single ventricle (mostly LV)	Hom	c.A2002T	p.I668F	6.05E-04	T	34
FGT07005391	M	European (ASJ)	Yes	Single ventricle, Tricuspid atresia, Pulmonary atresia	Hom	c.A2002T	p.I668F	6.05E-04	T	34
#1-03679	M	European	No	Ebstein's disease, PFO, Pulmonary atresia	CompHet	c.C2236T/c.A2002T	p.R746C/p.I668F	4.78E-05/6.05E-04	T/T	33/34
#1-05696	F	Undetermined	Yes	Ventricular septum aneurysm, Hypoplastic left/right PA, Hypoplastic RV, Hypoplastic tricuspid valve, Single ventricle (mostly LV)	CompHet	c.C2098T/c.C2023T	p.R700C/p.R675W	3.98E-05/4.78E-05	T/T	35/35
FGT07007232	M	African	No	Single ventricle, Pulmonary atresia with intact ventricular septum	Inferred CompHet	c.G2750A/c.G2099A	p.R917H/p.R700H	1.59E-05/1.59E-05	D/T	35/35
*SMVB8866	F	European	Yes	Single left ventricle, Tricuspid atresia	Hom	c.A2681C	p.Y894S	2.39E-04	T	28
* #1-04102	F	Mexican	No	ASD, Ebstein's disease	Hom	c.T2452C	p.Y818H	4.78E-05	T	23

*The p.Y894S variant identified in SMVB8866 and the p.Y818H variant identified in 1-04102 do not pass the damaging filter (MetaSVM"D" or CADD≥30) but they are moderately deleterious with a CADD score above 20 and the patients presented consistent right-sided valvular defects. #Variants also reported at Lahrouchi et al (doi: 10.1172/JCI142148.).

Table S10. *H6PD* recessive genotypes and phenotypes.

ID	Sex	Ancestry	Consanguin-eous?	Phenotype	Diagnosis	Mutation Type	Gene	Position (GRCh37)	cDNA Change	Amino Acid Change	gnomAD WES Freq	gnomAD WGS Freq	Bravo Freq	Meta SVM	CADD
1-02854	M	Undetermined	No	CTD	Coarctation of the aorta, simple (isolated) | Coronary artery dilation or ectasia | Left superior vena cava connecting to the coronary sinus | Tricuspid regurgitation neonatal | Ventricular septal defect, membranous	CompHet	*H6PD*	1-9305024-T-G	c.T31G	p.L11V	8.53E-05	2.00E-04	1.04E-04	D	0.001
								1-9322373-C-T	c.C1001T	p.T334M	3.00E-04	4.00E-04	5.34E-04	D	34
								1-9323730-G-T	c.G1178T	p.C393F	6.11E-05	9.70E-05	3.03E-04	D	24.9
1-04921	F	European	No	CTD	Hypoplastic right ventricle (subnormal cavity volume) | Pulmonary atresia, intact ventricular septum	CompHet	*H6PD*	1-9323895-C-T	c.C1343T	p.A448V	8.00E-04	6.00E-04	8.76E-04	D	0.689
								1-9324047-C-T	c.C1495T	p.R499C	3.25E-05	.	7.96E-06	D	31
1-07090	F	European	Yes	OTH	Ventricular septal defect, muscular, trabecular	Hom	*H6PD*	1-9323957-C-T	c.C1405T	p.R469W	8.13E-06	.	.	D	28
FGT07005181	F	European	No	HTX	Single Ventricle | Heterotaxia syndrome | Double outlet right ventricle with atrioventricular canal defect | Polysplenia	ICH	*H6PD*	1-9323768-G-A	c.G1216A	p.D406N	2.00E-04	9.70E-05	1.11E-04	D	1.664
								1-9324084-T-C	c.T1532C	p.L511S	8.12E-06	6.46E-05	1.59E-05	D	25.5
FGT07007142	M	European	No	OTH	Single Ventricle | Triscuspid atresia | D-loop, No transposition of the great arteries (Type 1) | Pulmonary atresia (Type 1a)	ICH	*H6PD*	1-9322373-C-T	c.C1001T	p.T334M	3.00E-04	4.00E-04	5.34E-04	D	34
								1-9324104-G-A	c.G1552A	p.G518S	1.00E-04	3.00E-04	1.75E-04	D	2.013
S8483H92	F	Mexican	No	OTH (AVC)	AV septal defect (include isolated primum ASD or inlet VSD in above categories)|Atrioventricular septal defect	ICH	*H6PD*	1-9322333-C-T	c.C961T	p.R321C	3.00E-04	3.00E-04	2.95E-04	D	35
								1-9322373-C-T	c.C1001T	p.T334M	3.00E-04	4.00E-04	5.34E-04	D	34

Table S11. *TNS1* recessive genotypes and phenotypes.

ID	Sex	Ancestry	Consanguineous?	Phenotype	Diagnosis	Gene	Mutation Type	cDNA Change	Amino Acid Change	gnomAD WES Freq	gnomAD WGS Freq	Bravo Freq	Meta SVM	CADD
1-03161	M	Undetermined	No	OTH	Atrial septal defect, secundum | Congenital aortic valve abnormality | Dysplastic tricuspid valve | Left aortic arch with normal branching pattern | Right atrial dilatation | Right ventricular hypertrophy	*TNS1*	CompHet	c.A3968G/c.G779C	p.Y1323C/p.R260P	2.00E-04/1.00E-03	8.00E-04/9.00E-04	7.25E-04/8.76E-04	D/D	15.6/32.0
1-07058	F	European	No	OTH	Aberrant right subclavian artery | Atrial septal defect, secundum | Patent ductus arteriosus restrictive	*TNS1*	CompHet	c.C2755T/c.G2681A	p.R919C/p.R894Q	3.66E-05/3.26E-05	6.47E-05/3.23E-05	3.19E-05/2.39E-05	D/D	35.0/33.0
1-16569	M	European	No	CTD (TOF)	Hypoplastic pulmonary annulus | Left superior vena cava connecting to the coronary sinus | Patent foramen ovale | Pulmonary artery stenosis | Tetralogy of Fallot | Ventricular septal defect, malalignment	*TNS1*	CompHet	c.C1559T/c.G325A	p.S520F/p.V109I	8.13E-06/6.00E-04	0/7.00E-04	0/3.42E-04	D/D	24.5/23.6
BRZ-2700	F	Undetermined	Yes	CTD (TOF)	Tetralogy of Fallot	*TNS1*	Hom	c.A3968G	p.Y1323C	2.00E-04	8.00E-04	7.25E-04	D	15.6
GT04011752	M	Undetermined	No	LVO (HLHS)	HYPOPLASTIC LEFT HEART SYNDROME;MITRAL ATRESIA;AORTIC ATRESIA	*TNS1*	CompHet	c.C3904T/c.G3442A	p.R1302C/p.D1148N	8.21E-05/5.00E-04	9.69E-05/5.00E-04	4.78E-05/7.33E-04	D/D	32.0/25.7
S29JA667	F	European	No	CTD (TOF)	Tetralogy of Fallot or DORV or pulmonary atresia|Tetralogy of Fallot or DORV	*TNS1*	Hom	c.G779C	p.R260P	1.00E-03	9.00E-04	8.76E-04	D	32

Table S12. 152 genes harbor more than one recessive genotype.

Gene	# RGs	pRec	Heart expr %rank	Binomial P-Val
**GDF1**	13	0.55	NA	1.82E-28
C1orf127	5	0.56	35.2	1.46E-10
**MYH6**	12	0.71	100.0	1.40E-09
**PLD1**	6	0.86	51.4	3.24E-09
H6PD	6	0.91	70.4	1.90E-07
VPREB3	2	0.59	16.6	3.70E-06
CD36	3	0.00	84.7	6.05E-06
USP17L7	2	NA	NA	1.62E-05
TNS1	6	0.94	98.6	4.42E-05
**DNAI2**	3	0.69	22.0	4.99E-05
**DNAH5**	7	1.00	0.0	8.90E-05
ZNF844	2	0.52	NA	9.27E-05
UNC45B	3	0.85	97.0	1.72E-04
KCNJ12	3	0.87	45.1	2.54E-04
DAW1	2	0.99	16.8	4.46E-04
EPB41L4A	3	0.11	61.0	5.51E-04
C15orf52	2	0.17	42.7	6.01E-04
CFTR	4	0.00	24.0	6.37E-04
HP	2	0.89	20.0	7.45E-04
MYO18B	5	1.00	97.8	7.89E-04
PCDHB1	2	0.14	0.0	9.77E-04
TRAF2	2	0.00	57.3	9.99E-04
HEPHL1	4	0.00	10.3	1.03E-03
LRP5L	2	0.02	NA	1.10E-03
TRPM2	3	0.00	18.3	1.23E-03
OTOL1	2	0.00	10.3	1.28E-03
DYSF	5	1.00	85.6	1.32E-03
PPEF2	2	0.65	16.3	1.33E-03
SLC25A31	2	0.14	37.8	1.34E-03
CTSC	2	0.80	74.5	1.42E-03
ERCC2	3	0.05	62.4	1.64E-03
ABCA7	5	0.00	54.2	1.94E-03
ALS2CL	2	0.00	35.3	2.07E-03
CRB2	3	0.92	49.3	2.13E-03
MMACHC	2	0.01	69.8	2.15E-03
ACOX2	2	0.97	0.0	2.23E-03
KEL	2	0.03	35.2	2.32E-03
ATP2C2	3	0.00	26.8	2.39E-03
KRT35	2	0.92	0.0	2.61E-03
ABCB5	3	0.00	14.2	2.81E-03
CAPN14	2	0.02	NA	2.92E-03
KRT72	2	0.10	0.0	2.96E-03
ABCC2	3	0.00	16.1	3.50E-03
ANKRD36	2	0.00	NA	3.54E-03
PCK2	2	0.00	80.9	3.69E-03
SVIL	3	1.00	96.4	3.89E-03
**ATIC**	2	0.01	79.8	3.96E-03
TMPRSS15	2	0.00	11.1	4.01E-03
ATAD3C	2	0.46	81.8	4.38E-03
HFM1	2	0.08	27.2	5.06E-03
GJA9	2	0.03	NA	5.15E-03
MFGE8	2	0.96	89.2	5.18E-03
HEXA	2	0.77	74.6	5.23E-03
PROC	2	0.98	0.0	5.47E-03
MYO15A	6	0.91	16.1	5.58E-03
MYO7A	5	0.61	47.7	5.68E-03
TG	3	0.00	39.7	5.86E-03
LEF1	2	0.00	34.5	6.13E-03
SPG11	3	0.95	56.2	6.47E-03
SCN5A	5	0.09	92.5	6.69E-03
ALDH1L2	2	0.73	78.9	7.15E-03
**NADSYN1**	2	0.68	45.6	7.27E-03
DGCR2	2	0.97	87.9	7.50E-03
DNAH7	4	0.03	35.8	9.06E-03
SLC26A4	2	0.01	16.7	9.40E-03
COL6A2	3	1.00	94.9	9.52E-03
PAPLN	2	0.00	32.7	9.59E-03
ALPI	2	0.00	10.3	1.08E-02
ATP13A4	2	0.16	10.3	1.20E-02
**PKD1L1**	2	1.00	15.5	1.21E-02
COL21A1	2	0.06	NA	1.21E-02
PCDH15	2	0.99	29.0	1.21E-02
KCNH6	3	0.00	23.6	1.24E-02
DSTYK	2	0.49	62.0	1.35E-02
TRPM4	2	0.00	64.8	1.43E-02
PKHD1L1	4	0.00	52.1	1.68E-02
ADAMTS18	2	0.14	21.5	1.79E-02
MYO1A	2	0.00	30.4	1.85E-02
COL9A2	2	1.00	54.8	1.92E-02
ECE2	2	0.11	44.8	1.99E-02
TMPRSS9	2	0.48	12.5	2.25E-02
MYBPC3	2	1.00	99.8	2.46E-02
VWA8	2	0.00	80.2	2.48E-02
NRP2	2	1.00	70.6	2.58E-02
ADAMTSL1	2	1.00	51.7	2.63E-02
MYH4	3	0.00	53.9	2.67E-02
ZFHX3	3	0.00	80.8	2.79E-02
THBS3	2	0.51	43.7	2.85E-02
OBSCN	6	0.00	98.2	2.90E-02
TNC	2	1.00	65.5	2.92E-02
ABCC12	2	0.00	14.6	3.27E-02
MYO7B	3	1.00	20.7	3.27E-02
ABCB11	2	1.00	9.5	3.34E-02
NOTCH1	4	0.00	87.9	3.50E-02
COL1A1	3	0.00	98.9	3.68E-02
FBN3	4	1.00	NA	4.21E-02
COL18A1	2	1.00	98.2	4.25E-02
USH2A	3	0.00	15.4	4.77E-02
APC	3	0.00	84.9	5.06E-02
HKDC1	2	0.92	18.0	5.15E-02
PIEZO1	2	1.00	93.8	5.20E-02
CACNA1H	5	1.00	92.7	5.38E-02
DNAH3	3	0.86	13.6	5.56E-02
ZNFX1	2	0.00	65.6	5.59E-02
ATP2B4	2	1.00	81.8	6.08E-02
MYH11	3	0.23	35.5	6.30E-02
**DNAH9**	3	1.00	18.4	6.81E-02
ATP7B	2	0.00	36.0	7.36E-02
OTOG	2	1.00	NA	7.42E-02
CPS1	2	1.00	0.0	7.89E-02
SCN7A	2	1.00	37.5	7.90E-02
COL5A2	2	0.00	95.7	9.79E-02
LAMA1	2	1.00	40.0	1.02E-01
PTCH1	2	0.00	70.9	1.04E-01
RYR3	7	0.29	76.7	1.10E-01
LAMA2	2	1.00	87.5	1.12E-01
CACNA1G	4	0.00	65.7	1.22E-01
TENM4	3	0.00	62.8	1.29E-01
COL4A1	2	0.00	99.6	1.31E-01
MYH1	2	0.00	55.3	1.31E-01
VWF	2	1.00	84.5	1.31E-01
KCNH3	2	0.00	24.7	1.38E-01
MYH8	2	0.00	53.1	1.41E-01
STAB1	2	0.93	93.2	1.53E-01
OTOF	2	0.22	26.8	1.54E-01
ADGRV1	2	1.00	33.5	1.56E-01
SCN10A	2	0.00	43.9	1.72E-01
NEB	4	1.00	39.1	1.77E-01
MYH3	2	1.00	34.8	1.89E-01
FLNB	3	1.00	86.7	1.89E-01
COL5A1	2	0.00	97.2	2.00E-01
**DYNC2H1**	2	1.00	44.4	2.05E-01
**DNAH11**	2	1.00	NA	2.09E-01
PLEC	5	1.00	98.1	2.34E-01
CACNA1B	3	0.00	22.8	2.54E-01
ACACB	2	0.19	89.4	2.64E-01
LRP5	2	0.49	75.5	2.66E-01
DNAH10	2	1.00	22.3	2.78E-01
RYR1	8	1.00	27.2	3.34E-01
UBR4	2	0.00	94.9	3.45E-01
COL7A1	2	1.00	34.5	3.46E-01
FBN2	2	0.00	97.8	4.46E-01
SYNE1	3	1.00	87.1	4.53E-01
HMCN1	2	1.00	85.9	4.80E-01
HSPG2	2	1.00	99.6	4.86E-01
TTN	22	1.00	99.9	5.77E-01
CACNA1A	2	0.00	48.7	5.81E-01
MACF1	2	0.00	97.9	6.41E-01
DST	2	0.00	93.2	6.86E-01
LRP1B	2	1.00	9.5	7.05E-01
LRP1	3	0.00	92.9	8.96E-01
RYR2	2	0.00	98.5	9.28E-01

The genes were sorted by binomial p-values; Known human recessive CHD genes were bolded; pRec: probability of being intolerant to biallelic LoF variants based on gnomAD dataset (doi: 10.1038/nature19057); Heart expr %rank: the percentile of gene expression in mouse heart at embryonic day E14.5 (doi: 10.1038/nature12141).

Table S13. Number of genes with ≥ 2 damaging recessive genotypes is higher than expected.

Gene class	# Genes observed	# Genes expected	Enrichment [95% CI]	Permutation P-value
**All genes**				
**Genes with ≥ 2 RGs**				
LoF	9	1.1	7.92 [2.25, inf]	**2.00E-06**
Damaging	152	117.8	1.29 [1.13, 1.49]	**2.30E-05**
Syn	578	580.6	1.00 [0.95, 1.05]	0.58
**After excluding 108 known human recessive CHD genes**				
**Genes with ≥ 2 RGs**				
LoF	8	1.0	8.28 [2.67, inf]	**6.00E-06**
Damaging	141	106.5	1.32 [1.16, 1.55]	**1.90E-05**
Syn	568	563.4	1.01 [0.96, 1.07]	0.39

Table S14. Enrichment analysis for RGs in genes specifically expressed in notochord or cardiomyocytes.

(A) Enrichment analysis of RGs in 450 notochord-specific genes

Sample set	# RGs	# Observed RGs in 450 notochord-specific genes	# Expected RGs in 450 notochord-specific genes	Enrichment	P-value
All 5424 cases	1253	72	27.90	2.58	1.11E-12
1160 Laterality-associated defect cases	300	23	6.51	3.53	2.57E-07
4134 Non-laterality-associated defect cases	939	49	21.04	2.33	8.99E-08
1798 Autism controls	229	4	5.08	0.79	0.75

(B) Enrichment analysis of RGs in 150 cardiomyocyte-specific genes

Sample set	# RGs	# Observed RGs in 150 cardiomyocyte-specific genes	# Expected RGs in 150 cardiomyocyte-specific genes	Enrichment	P-value
All 5424 cases	1253	58	41.76	1.39	8.90E-03
1849 Left-sided defect cases	397	31	17.12	1.81	1.24E-03
3445 Non-left-sided defect cases	842	26	23.31	1.12	0.31
1798 Autism controls	229	9	8.73	1.03	0.51

The 130 CHD cases without subtype information are not included in the enrichment analysis

Table S15. Estimation of the fraction of recessive genotype in consanguineous vs nonconsanguineous cases.

Type of RGs	# Expected RGs in Parents	# Observed RGs in Parents	# Observed / # Expected in Parents	# Expected RGs in Probands	# Observed RGs in Probands	# Observed / # Expected in Probands	Fraction of Probands with Causal RGs	Confidence Interval
**414 Consanguineous Trios (414 probands, 828 parents)**								
Syn	1043.9	1045	1.00	673.2	671	1.00	-	-
LoF	50.4	37	0.73	36.8	43	1.17	3.82%	[0.75%,6.88%]
Damaging	368.5	320.0	0.87	248.0	245	0.99	6.88%	[-0.24%,14.01%]
**3302 Non-Consanguineous Trios (3302 probands, 6604 parents)**								
Syn	3083.6	3090	1.00	1280.1	1280	1.00	-	-
LoF	102.0	58	0.57	29.8	18	0.60	0.03%	[-0.22%,0.28%]
Damaging	1035.0	861	0.83	399.4	385	0.96	1.58%	[0.43%,2.73%]

Table S16. Estimation of the fraction of recessive genotype in cases with laterality-associated defect.

Type of RGs	# Expected RGs in Parents	# Observed RGs in Parents	# Observed / # Expected in Parents	# Expected RGs in Probands	# Observed RGs in Probands	# Observed / # Expected in Probands	Fraction of Probands with Causal RGs	Confidence Interval
**783 Laterality-Associated Defect Trios (783 probands, 1566 parents)**								
Syn	967.7	967	1.00	482.2	484	1.00	-	-
LoF	40.7	19	0.47	20.7	22	1.06	1.56%	[0.40%,2.73%]
Damaging	344.0	267	0.78	171.3	176	1.03	5.36%	[2.12%,8.61%]
**2923 Non-Laterality-Associated Defect Trios (2923 probands, 5846 parents)**								
Syn	3170.2	3159	1.00	1464.6	1462	1.00	-	-
LoF	117.2	76	0.65	49.2	39	0.79	0.24%	[-0.18%,0.66%]
Damaging	1080.8	913	0.84	486.2	451	0.93	1.36%	[-0.05%,2.77%]

Trios without phenotype information are not included

Table S17. Estimation of the recessive genotype contribution among Ashkenazim.

Type of RGs	# Expected RGs in Parents	# Observed RGs in Parents	# Observed / # Expected in Parents	# Expected RGs in Probands	# Observed RGs in Probands	# Observed / # Expected in Probands	Fraction of Probands with Causal RGs	Confidence Interval
**279 AJ Trios (279 probands, 558 parents)**								
Syn	260.2	260	1.00	121.5	121	1.00	-	-
LoF	10.7	10	0.93	4.6	6	1.30	0.60%	[-1.12%,2.32%]
Damaging	98.2	94	0.96	45.2	56	1.24	4.55%	[-0.69%,9.79%]
**3437 Non-AJ Trios (3437 probands, 6874 parents)**								
Syn	3874.3	3875	1.00	1826.6	1830	1.00	-	-
LoF	146.1	85	0.58	64.5	55	0.85	0.51%	[0.09%,0.93%]
Damaging	1323.0	1087	0.82	611.1	574	0.94	2.06%	[0.71%,3.40%]

Dataset S1 (separate file). Clinical description of CHD probands.

Dataset S2 (separate file). Recessive genotypes in 5,424 CHD cases.

Dataset S3 (separate file). List of known human recessive CHD genes.

SI References

1. N. Krumm *et al.*, Excess of rare, inherited truncating mutations in autism. *Nat Genet* **47**, 582-588 (2015).
2. S. C. Jin *et al.*, Contribution of rare inherited and de novo variants in 2,871 congenital heart disease probands. *Nat Genet* **49**, 1593-1601 (2017).
3. A. McKenna *et al.*, The Genome Analysis Toolkit: a MapReduce framework for analyzing next-generation DNA sequencing data. *Genome Res* **20**, 1297-1303 (2010).
4. G. A. Van der Auwera *et al.*, From FastQ data to high confidence variant calls: the Genome Analysis Toolkit best practices pipeline. *Curr Protoc Bioinformatics* **43**, 11 10 11-11 10 33 (2013).
5. E. Garrison, G. Marth, Haplotype-based variant detection from short-read sequencing. *arXiv* https://arxiv.org/abs/1207.3907, 1207.3907 (2012).
6. S. C. Jin *et al.*, Exome sequencing implicates genetic disruption of prenatal neuro-gliogenesis in sporadic congenital hydrocephalus. *Nat Med* **26**, 1754-1765 (2020).
7. K. J. Karczewski *et al.*, The mutational constraint spectrum quantified from variation in 141,456 humans. *Nature* **581**, 434-443 (2020).
8. D. Taliun *et al.*, Sequencing of 53,831 diverse genomes from the NHLBI TOPMed Program. *bioRxiv* 10.1101/563866, 563866 (2019).
9. C. Dong *et al.*, Comparison and integration of deleteriousness prediction methods for nonsynonymous SNVs in whole exome sequencing studies. *Hum Mol Genet* **24**, 2125-2137 (2015).
10. P. Rentzsch, D. Witten, G. M. Cooper, J. Shendure, M. Kircher, CADD: predicting the deleteriousness of variants throughout the human genome. *Nucleic Acids Res* **47**, D886-D894 (2019).
11. S. R. Browning, B. L. Browning, Rapid and accurate haplotype phasing and missing-data inference for whole-genome association studies by use of localized haplotype clustering. *Am J Hum Genet* **81**, 1084-1097 (2007).
12. S. Purcell *et al.*, PLINK: a tool set for whole-genome association and population-based linkage analyses. *Am J Hum Genet* **81**, 559-575 (2007).
13. A. L. Price *et al.*, Principal components analysis corrects for stratification in genome-wide association studies. *Nat Genet* **38**, 904-909 (2006).
14. M. Lemaire *et al.*, Recessive mutations in DGKE cause atypical hemolytic-uremic syndrome. *Nat Genet* **45**, 531-536 (2013).
15. C. Wang *et al.*, Ancestry estimation and control of population stratification for sequence-based association studies. *Nat Genet* **46**, 409-415 (2014).
16. S. M. Bray *et al.*, Signatures of founder effects, admixture, and selection in the Ashkenazi Jewish population. *Proc Natl Acad Sci U S A* **107**, 16222-16227 (2010).
17. H. C. Martin *et al.*, Quantifying the contribution of recessive coding variation to developmental disorders. *Science* **362**, 1161-1164 (2018).
18. K. E. Samocha *et al.*, A framework for the interpretation of de novo mutation in human disease. *Nat Genet* **46**, 944-950 (2014).
19. B. Pijuan-Sala *et al.*, A single-cell molecular map of mouse gastrulation and early organogenesis. *Nature* **566**, 490-495 (2019).
20. S. Durinck, P. T. Spellman, E. Birney, W. Huber, Mapping identifiers for the integration of genomic datasets with the R/Bioconductor package biomaRt. *Nat Protoc* **4**, 1184-1191 (2009).
21. V. Narasimhan *et al.*, BCFtools/RoH: a hidden Markov model approach for detecting autozygosity from next-generation sequencing data. *Bioinformatics* **32**, 1749-1751 (2016).
